# DMH1-loaded peptide nanomicelles restore myelin and attenuate neuroinflammation in trigeminal neuralgia via CCL5 suppression

**DOI:** 10.3389/fphar.2025.1590624

**Published:** 2025-08-06

**Authors:** Shuangyin Xia, Xiunan Qin, Yaping Wang

**Affiliations:** ^1^ Department of Anesthesiology, The Second Xiangya Hospital, Central South University, Changsha, China; ^2^ Department of Pain Management, The Second Xiangya Hospital, Central South University, Changsha, China; ^3^ Hunan Province Center for Clinical Anesthesia and Anesthesiology, Research Institute of Central South University, Changsha, China; ^4^ Clinical Research Center for Pain Medicine in Hunan Province, Changsha, China

**Keywords:** peptide nanobundles, bone morphogenetic protein 4, C-C motif chemokine ligand 5, oligodendrocyte progenitor cells, demyelinating lesions, trigeminal neuralgia, inflammation suppression

## Abstract

**Background:**

Trigeminal neuralgia (TN), a debilitating neuropathic pain disorder, is characterized by demyelination and neuroinflammation, with limited therapies addressing its underlying pathophysiology. Bone Morphogenetic Protein 4 (BMP4) signaling and chemokine CCL5 are implicated in neuroinflammation and oligodendrocyte dysfunction, presenting potential therapeutic targets.

**Methods:**

Peptide nanomicelles loaded with the BMP4 inhibitor DMH1 (NM@DMH1) were synthesized and characterized for stability, drug release kinetics, and biocompatibility. *In vitro* studies assessed oligodendrocyte progenitor cell (OPC) differentiation and anti-inflammatory effects in lipopolysaccharide-induced models. A rat TN model (chronic infraorbital nerve compression) evaluated NM@DMH1’s efficacy in alleviating mechanical allodynia, demyelination, and neuroinflammation. Mechanistic roles of CCL5 were explored using recombinant protein supplementation.

**Results:**

NM@DMH1 exhibited uniform nanostructure (120 nm), high encapsulation efficiency (82%), and pH-responsive sustained release. Treatment enhanced OPC differentiation, reduced pro-inflammatory cytokines (IL-6, TNF-α, IL-1β), and suppressed CCL5 expression *in vitro*. In TN rats, NM@DMH1 significantly attenuated mechanical pain hypersensitivity (p < 0.01 vs model), restored myelin markers (MBP, MOG), and inhibited neuroinflammatory infiltration. CCL5 supplementation reversed therapeutic benefits, confirming its pivotal role.

**Conclusion:**

NM@DMH1 represents a nanotechnology-driven strategy targeting TN pathogenesis by promoting remyelination and suppressing CCL5-mediated neuroinflammation. This study advances precision drug delivery for neuropathic pain and highlights CCL5 as a novel therapeutic node, offering translational potential for TN and related neuroinflammatory disorders.

## Graphical Abstract

**Figure:**
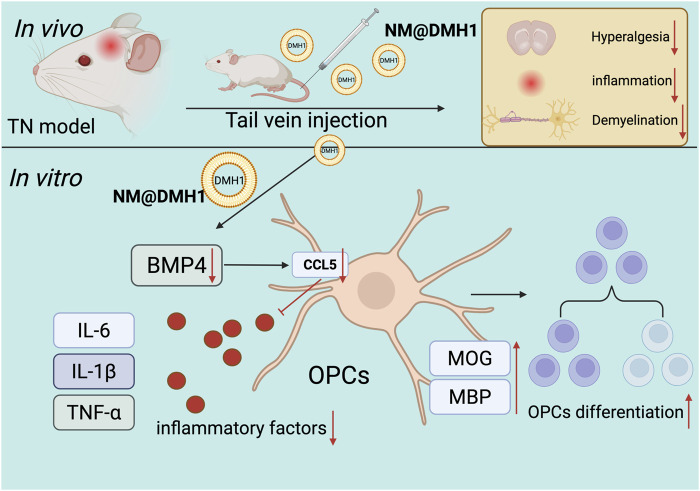
Molecular mechanisms of peptide nanobundles@DMH1 promoting differentiation in OPCs and its therapeutic implications for TN treatment.

## 1 Introduction

Trigeminal neuralgia (TN) is a prevalent chronic neuropathic pain (NP) considered to be among the most severe facial pains (Jones et al., 2019). It typically presents as sudden, shock-like, or intense burning pain, often triggered by mild facial touches, severely affecting patients’ quality of life and psychological health (Zakrzewska, 2002). Statistics indicate a global increase in the incidence of TN, with the risk escalating with age. This trend is likely linked to the degenerative changes in the nervous system associated with aging (Ayele et al., 2020). Although current treatments for TN include pharmacotherapy and surgery, these methods often provide only temporary relief rather than a cure. Moreover, approximately 10%–20% of patients may experience pain recurrence within several years. Additionally, these treatments carry high risks and costs, making them unsuitable for all patients (Lambru et al., 2021). Therefore, developing new and effective treatment strategies is crucial for enhancing the quality of life for patients.

The pathophysiology of TN is complex, primarily involving demyelination of the nerve root and localized inflammatory responses. Research indicates that vascular compression, particularly by the cerebellar arteries, is a leading cause of nerve root inflammation and demyelination (Prasetya et al., 2022). Demyelination refers to damage to the myelin sheath surrounding nerve fibers, which impedes neural signal transmission and triggers pain (Love, 2001). This demyelination is often caused by microvascular compression, infection, autoimmune diseases, or genetic factors (Liu et al., 2023). The inflammatory response results from the release of inflammatory mediators such as cytokines and chemokines during the tissue damage and repair process following demyelination. These mediators can activate or sensitize nerve fibers, exacerbating the pain (Yao et al., 2020; Ostertag et al., 2023). Understanding these mechanisms is crucial for designing effective interventions for the treatment of TN.

Studies have shown that central Bone Morphogenetic Protein 4 (BMP4) is elevated during NP, acting as an independent regulator of glial cells (Chen et al., 2024). Additionally, BMP4 plays a crucial role in astrocyte activation following spinal cord injury, where it may trigger glial activation through the p-Smad 1/5/8 and p-STAT3 signaling pathways, thereby promoting abnormal pain in rats (Yang et al., 2019). However, the clinical application of BMP4 inhibitor is limited by its rapid degradation *in vivo* and the challenges of precise targeted delivery (Zhang et al., 2019). Researchers have begun exploring nanotechnology as a delivery system to overcome these obstacles. In particular, peptide nanobundles, with their excellent biocompatibility and controlled release characteristics, have emerged as a promising platform (Chen et al., 2020; Yilgor et al., 2009). Using peptide nanotechnology can protect BMP4 inhibitor Dorsomorphin Homolog 1 (DMH1) from premature degradation and enable targeted delivery to specific neural cells by altering the chemical properties of the nanobundles (Peng et al., 2020).

Chemokines play a crucial role in host defense by regulating immune cell production, organizing their distribution across specific tissues under basal conditions, and controlling their recruitment and activation in response to inflammatory stimuli. C-C motif chemokine ligand 5 (CCL5, also known as RANTES) is a ligand for chemokine receptors CCR1, CCR3, and CCR5, and it guides the migration of monocytes/macrophages and T cells (Ajuebor et al., 2001; Bless et al., 2000). Extensive evidence suggests that CCL5 modulates inflammatory responses in various pathological conditions. Dysregulation of CCL5 activity can lead to disease and has been implicated in the pathogenesis of multiple inflammatory diseases (Jiang et al., 2020; Pawlik et al., 2021). CCL5 has also been identified in damaged nerves, indicating a potential role in neuropathology. Studies have shown that the absence of CCL5 modulates the recruitment of inflammatory cells to pain and inflammation sites, potentially alleviating pain in mouse models of chronic neuropathic pain (Liou et al., 2012). Moreover, BMP signaling has been found to regulate CCL5 expression in multiple sclerosis, influencing the recruitment of macrophages and T lymphocytes (Tang et al., 2022).

This study aims to validate the effectiveness of exogenous DMH1 peptide nanobundles in downregulating CCL5 expression, promoting the differentiation of oligodendrocyte progenitor cells (OPCs) and inhibiting demyelinating lesions in a rat model of TN. Through both *in vitro* and *in vivo* experiments, we will explore the biodistribution, cellular uptake, and regulatory effects of peptide nanobundles@DMH1 on inflammatory cytokines and neural markers. We anticipate our findings will elucidate the molecular mechanisms by which peptide nanobundles@DMH1 alleviates NP and confirm its potential as a novel therapeutic strategy for neurological disorders. This study provides an effective new treatment option for TN and theoretical and technical support for treating other NP conditions, with significant scientific and clinical implications.

## 2 Materials and methods

### 2.1 Synthesis of amphiphilic peptides

The synthesis of amphiphilic peptides was carried out as follows: Lys (Z)-NCA (1676-86-4, PMC ISOCHEM) was subjected to ring-opening polymerization (ROP) in anhydrous chloroform (CHCl_3_, 650498, Merck) at 40°C under a nitrogen atmosphere, with ethylenediamine (E26266, Merck) serving as the initiator. This process yielded poly (Nε-carbobenzoxy-L-lysine) (PZLL, P4510, Merck) after 3 days of reaction. The molar ratio of Lys (Z)-NCA to ethylenediamine was 30:1, and the concentration of Lys (Z)-NCA solution was 0.1 g/mL. After the reaction, the solvent chloroform (CHCl_3_) was removed under reduced pressure to concentrate the reaction mixture. PZLL was obtained by precipitation in cold diethyl ether, yielding 90%. Subsequently, using the same procedure, a block copolymer, poly (Nε-carbobenzoxy-L-lysine)-block-poly (L-phenylalanine) (PZLL-b-Phe), was synthesized with a yield of 72.5%. The mass ratio of PZLL to Phe-NCA (14825-82-2, PMC ISOCHEM) was 4:1, and the concentration of the Phe-NCA solution was 80 mg/mL. The third step involved the removal of the carbobenzoxy protective group from the PZLL block, converting PZLL-b-Phe to poly (L-lysine)-block-poly (L-phenylalanine) (PLL-b-Phe). Typically, 0.8 g of PZLL-b-Phe was dissolved in 12 mL of trifluoroacetic acid (T6508, Merck) and cooled in an ice-water bath. Subsequently, 2 mL of 33% hydrobromic acid-acetic acid solution (HBr-Hac, 248630, Merck) was added dropwise over 30 min with stirring at 4°C for 1 h, followed by stirring at room temperature for 3 h. The resulting PLL-b-Phe was precipitated in excess cold anhydrous ether (270989, Merck), dissolved in N, N-dimethylformamide (227056, Merck), dialyzed against distilled water (MWCO 3500 Da), and freeze-dried to yield a white solid with an 80% yield. Finally, mPEG-g-PLL-b-Phe was synthesized through a reductive amination reaction involving the formation of a Schiff base between the amino groups of PLL-b-Phe and the aldehyde groups of mPEG-CHO. Specifically, 0.4 g of PLL-b-Phe was dissolved in a mixture of dimethyl sulfoxide (DMSO, D2650, Merck) and water (1:1 volume ratio, 4 mL) and stirred while 0.5 mL of mPEG-CHO (125061-88-3, Avantor) aqueous solution (100 mg) was added dropwise. Subsequently, 6 mg of NaBH_3_CN was added in three equal portions at 6-h intervals. After 24 h of reaction, the solution was dialyzed against distilled water for 48 h and freeze-dried to obtain a light yellow solid, mPEG-g-PLL-b-Phe, referred to as NM. The ^1^H Nuclear Magnetic Resonance (NMR) spectra were recorded on a Bruker 400 MHz NMR spectrometer (Bruker) using CDCl_3_ or DMSO-D_6_ as solvents at room temperature (Rao et al., 2008).

### 2.2 Formation of nanobundles

Nanobundles were prepared using the dialysis method. Specifically, 30 mg of mPEG-g-PLL-b-Phe was dissolved in 16 mL of DMSO (D2650, Merck) and subjected to ultrasonic treatment. The solution was then dialyzed in deionized water (MWCO 3500 Da, PURX35015, Merck) for 24 h to remove the organic solvent, allowing micelle formation. The dialyzed suspension was freeze-dried to obtain the nanobundles.

### 2.3 Transmission electron microscopy (TEM)

The morphology of the nanobundles was studied using Cryo-SEM high-pressure freezing techniques (EM Cryo CLEM, Leica), which prevent or minimize structural damage caused by ice crystal formation. Samples (5 µL) were placed on a cold sample holder and transferred to a liquid nitrogen bath until the nitrogen stopped boiling. The liquid nitrogen bath was then transferred to a vacuum space, where the samples were immediately subjected to vacuum. The samples were heated to −100°C and freeze-dried for 30 min by water sublimation. Observations were made at −146°C under an accelerating voltage of 3 kV or 5 kV using analytical mode and backscattered electron signals.

A 1 mm thick rat spinal cord sample was fixed with 2% glutaraldehyde for 1 h at 4°C for protein fixation. The sections were then rinsed with cacodylate buffer for 10 min, followed by fixation in a 0.1 M cacodylate buffer containing 1% osmium tetroxide and 1.5% potassium ferrocyanide at 4°C for 1 h. The samples were washed with Milli-Q water for 10 min and stained with 2% uranyl acetate for 30 min. Dehydration was performed using an increasing ethanol gradient. Prior to embedding, the samples were rinsed three times with propylene oxide at room temperature, each for 20 min. The samples were then infiltrated with a 1:1 mixture of propylene oxide and Spurr resin for 4–5 h, followed by infiltration with a 1:3 mixture for 15–16 h. Finally, the samples were infiltrated with 100% Spurr resin for 5–6 h. The samples were polymerized at 60°C for at least 16 h, after which they were placed in plastic molds to harden into resin blocks. Using a Leica Ultracut T ultramicrotome (Leica Microsystems AG, Wetzlar, Germany), 70 nm thick spinal cord cross-sections were cut. The thin sections were stained with 2% uranyl acetate for 12 min, followed by Reynolds’ lead citrate staining for 6 min, and prepared for TEM imaging (Touitou et al., 2018).

### 2.4 Dynamic light scattering (DLS) for particle size distribution

The physicochemical parameters of the nanobundles were characterized using a Malvern Zetasizer Nano Series DTS 1060 (Malvern Instruments SA, Worcestershire, United Kingdom). The hydrodynamic diameter (nm) and polydispersity index (PDI) were measured for each analysis. The nanomicelles were diluted 1/30 (v/v) in MilliQ purified water and measured three times at 25°C. Results were presented as the average size (nm) and size distribution based on intensity percentage (Touitou et al., 2018).

### 2.5 Differential scanning calorimetry (DSC)

DSC measurements were performed using a PerkinElmer DSC 8000 (PerkinElmer Instruments) at a heating rate of 10°C per minute unless otherwise specified. Approximately 5 mg of the sample was typically heated above its melting point to eliminate thermal history, followed by two heating/cooling cycles to ensure the reproducibility of the results (de Souza et al., 2025).

### 2.6 Thermogravimetric analysis (TGA)

TGA was conducted under a nitrogen atmosphere using the Mettler Toledo STARe system (DSC 5+, METTLER TOLEDO). The temperature range was set from 0°C to 550°C, with a scanning rate of 10°C per minute and a sample weight of 10–12 mg (Slavkova et al., 2025).

### 2.7 Preparation of DMH1-Loaded nanobundles

BMP4 inhibitor DMH1-loaded nanomicelles were prepared using a dialysis method. In brief, DMH1 (53295ES10, YeaSen Biotechnology) was purchased from YeaSen Biotechnology. A mixture of 30 mg mPEG-g-PLL-b-Phe, 10 mg DMH1, and 2 µL triethylamine (T0886, Merck) was dissolved in 16 mL DMSO and subjected to ultrasonic treatment. The solution was then dialyzed in deionized water (MWCO 3500 Da) for 24 h to remove organic solvents and unbound DMH1, allowing micelle formation. The resulting DMH1-loaded nanobundles were designated as NM@DMH1.

### 2.8 Encapsulation efficiency (EE) measurement

The EE of DMH1 in the nanobundles was indirectly measured using a centrifugal ultrafiltration method. An Amicon Ultra-2 centrifugal filter device (UFC205024, Millipore) with a molecular weight cutoff (MWCO) of 100 kDa and a centrifugal force of 7,500 × g was used to remove unencapsulated DMH1. The UV absorbance of the filtrate was measured at 330 nm for 10 min. The EE of DMH1 in the nanobundles was calculated by comparing the total amount of DMH1 used in the hydration step (Drug_Total_) to the amount of free DMH1 in the supernatant (Drug_Free_) using the following equation (Slavkova et al., 2025):

$$\text{EE}\%=\left[\left({\text{Drug}}_{\text{Total}}-{\text{Drug}}_{\text{Free}}\right)\;/\;{\text{Drug}}_{\text{Total}}\right]\times 100\%$$

### 2.9 Drug release study

The *in vitro* release of DMH1 from nanomicelles was evaluated using a dialysis bag diffusion technique (FDM314, Beyotime, MWCO 14 kDa). Six milligrams of DMH1-loaded nanomicelles were re-dispersed in 6 mL of DEPC water using brief sonication and then divided into two equal portions. Each sample was sealed in a pre-treated dialysis bag and immersed separately in 60 mL of phosphate-buffered saline (PBS) buffer (pH = 7.4 or 5.0). The system was gently shaken at 37°C and kept in the dark. At predefined time intervals, 3 mL samples were withdrawn from the medium outside the dialysis bag for fluorescence measurement, with an equal volume of fresh buffer added to maintain the system volume. The release of DMH1 was quantified by high-performance liquid chromatography (HPLC) at a detection wavelength of 340 nm. The cumulative drug release was calculated and expressed as a percentage over time (Suo et al., 2016).

### 2.10 Long-term storage stability

The nanobundles were stored at 4°C ± 2°C and protected from light. The particle size was characterized at specified time points using DLS to assess stability.

### 2.11 Circular dichroism (CD) spectroscopy

CD measurements were conducted using a Jasco J-715 spectropolarimeter with a Peltier temperature controller. Far-ultraviolet CD was employed to characterize protein folding/unfolding and thermal stability. Samples were prepared at approximately 0.075 mg/mL, dissolved in 50 mM potassium phosphate (pH 6.0 and 7.0, respectively) and 100 mM NaCl. Scanning was performed at 25°C, with a scan speed of 10 nm/min across a wavelength range of 190–260 nm, using a quartz cuvette with a path length of 0.1 cm. To assess thermal stability, ellipticity changes at 222 nm were monitored across a temperature range of 5°C–100°C, with a heating rate of 0.5°C/min.

### 2.12 Isolation and identification of OPCs

OPCs were isolated from Sprague Dawley (SD) rats (Slac, SLAC Laboratory Animal Co., Ltd.). The procedure involved isolating neural stem cells from the hippocampal region of embryonic day 14 (E14) rats. These cells were cultured for 7 days in DMEM/F12 (11320033, Gibco) supplemented with 2% B27 (17504044, Gibco), 1% N2 supplement (17502048, Gibco), 20 ng/mL EGF (PHG0311, Gibco), and bFGF (100-18B-50UG, Gibco).

To generate OPCs, neural spheres were dissociated using StemPro Accutase (00-4555-56) and then plated onto uncoated culture dishes. The cells were cultured in an oligodendrocyte medium (DMEM/F12 with 2% B27) supplemented with 10 ng/mL bFGF and PDGF-AA growth factors (PHG0035, Gibco). Small adherent oligodendrocyte precursor clusters were formed. After two passages, the cells maintained a high enrichment of OPCs.

To assess the phenotype of the obtained oligodendrocyte cell line, cells on coverslips were fixed with 4% paraformaldehyde (P0099, Beyotime) at 4°C for 20 min, permeabilized with 0.1% Triton X-100 (P0096, Beyotime) for 15 min, and blocked with 5% goat serum (C0265, Beyotime) at 23°C for 60 min. The primary antibody against the oligodendrocyte marker OLIG2 (AF2418, R&D Systems) was diluted in 5% goat serum and incubated with the samples overnight at 4°C. The following day, the cells were incubated with the secondary antibody against rabbit IgG (H + L), F (ab') 2 fragment (4,412, CST) at 37°C for 1 h. Nuclei were stained with DAPI (C1002, Beyotime) at 23°C for 10 min. Fluorescent images were captured using a fluorescence microscope (Olympus Corporation, Tokyo, Japan; magnification ×200).

Cells were treated with 200 µL of lipopolysaccharide (LPS) at a concentration of 15 μg/mL for 24 h, followed by incubation with 200 µL of 20 μg/mL NM@DMH1 or DMH1 at 37°C for an additional 24 h. The experimental groups were as follows: PBS group: treated with PBS, LPS group: treated with 200 µL of LPS (15 μg/mL), LPS + DMH1 group: treated with 200 µL of LPS (15 μg/mL) followed by DMH1 (10 µM), LPS + NM group: treated with 200 µL of LPS (15 μg/mL) followed by 200 µL of NM; LPS + NM@DMH1 group: treated with 200 µL of LPS (15 μg/mL) followed by NM@DMH1 (10 µM) treatment (Sun et al., 2023).

### 2.13 CCK-8 assay

Cell toxicity was assessed using the CCK-8 assay (C0037, Beyotime). MC3T3-E1 cells were seeded at 4 × 10^3^ cells per well in a 96-well plate containing 100 μL of medium. After a 24-h incubation, cells were treated according to experimental groups. Following a 20-h incubation at 37°C, 10 µL of CCK-8 solution was added to each well. After 120 min of incubation, the optical density (OD) at 450 nm was measured (Jian et al., 2020).

### 2.14 Cell uptake experiment

To prepare the fluorescein isothiocyanate (FITC)-loaded micelle solution, 16 mg of FITC was added to 16 mL of DMSO micelle solution and sonicated for 30 min. The solution was centrifuged at 5,000 rpm for 10 min to remove unencapsulated FITC. Subsequently, 100 μL of the FITC-loaded micelle solution was diluted in 500 μL of DMEM and added to OPCs cultured in a 24-well plate. Cells were imaged at 0, 6, and 12 h using a fluorescence microscope (Olympus IX73^®^). Before imaging, the medium was removed, and cells were washed multiple times with PBS (Mammadova et al., 2025).

### 2.15 Flow cytometry

For quantitative analysis, flow cytometry was performed using the FACS Canto II™ cell sorter (BD Biosciences) and FACS Diva software (BD Biosciences) to study the uptake of FITC-labeled micelles by OPCs. Cells were plated in 35-mm dishes and incubated for 24 h. Following this, cells were incubated with 1 mL of FITC-labeled micelle solution (50 μg/mL) for 0, 6, and 12 h, washed with cold PBS, digested with trypsin, and centrifuged at 1,500 rpm. The cells were resuspended in PBS and analyzed for FITC intensity using flow cytometry.

To assess the inflammatory response in rat brain tissue after treatment with recombinant CCL5 protein, rats were anesthetized, and brain tissue was collected. The tissue was thoroughly dissociated by mechanical disruption, and single-cell suspensions were prepared by filtering through a 40 μm cell strainer. Immune cells were isolated using density gradient centrifugation (Percoll) to remove debris and other cell fragments, yielding high-purity immune cells. Antibodies were added and incubated at room temperature for 20 min, followed by two washes with PBS. The cells were then resuspended in PBS and analyzed for fluorescence intensity using flow cytometry. The following antibodies were used: Anti-CD3 (563948, 0.2 mg/mL, BD), Anti-CD4 (740513, 0.2 mg/mL, BD) to detect CD4^+^ T cells, Anti-CD11b (561691, 0.5 mg/mL, BD), and Anti-F4/80 (sc-52664, 0.2 mg/mL, Santa Cruz Biotechnology) to detect macrophages (Campanella et al., 2002; Misharin et al., 2013).

### 2.16 *In Vivo* distribution study

Optical images of FITC-labeled micelles were acquired using the *in vivo* imaging system (IVIS) Spectrum (Perkin Elmer Inc., MA). Free FITC and FITC-labeled micelle solutions (200 µL each) were injected into the tail veins of rats. *In vivo* near-infrared fluorescence imaging was performed at 0, 1, 12, and 24 h post-injection. Rats were anesthetized before imaging, and the IVIS Spectrum was used for optical scanning with FITC excitation (465 nm) and emission (520 nm) channels (Zhu et al., 2025).

### 2.17 Biocompatibility analysis

Twelve male Sprague Dawley (SD) rats (Slac:SD, Hunan Provincial Center for Laboratory Animals) were purchased and acclimated for 2 weeks prior to experimentation. At the time of the experiments, the rats were 8–10 weeks old and were randomly divided into two groups: the CTR group (administered 50 µL of PBS via tail vein injection) and the NM@DMH1 group (administered 50 µL of NM@DMH1 at a dose of 5 mg/kg via tail vein injection). Blood samples were collected from the tail vein on days 5, 14, and 28 post-injection, centrifuged at 1,500 × g for 10 min, and the supernatants were analyzed. Plasma levels of BUN (blood urea nitrogen), Scr (serum creatinine), ALT (alanine aminotransferase), AST (aspartate aminotransferase), and TBIL (total bilirubin) were measured using an automatic biochemical analyzer (BS-600, Shenzhen Mindray Biomedical Electronics Co., Ltd., Shenzhen, China).

On day 28 post-injection, rats were euthanized for biocompatibility assessment. Tissues from the heart, liver, spleen, lungs, and kidneys were fixed in a 4% formaldehyde solution, embedded in paraffin, and sectioned into 4 µm slices. Sections were stained with hematoxylin and eosin (H&E) using a staining reagent (C0105M, Beyotime) for 1 min, then rinsed with water until clear. The sections were subsequently counterstained with eosin for 15 s. After counterstaining, the sections were immediately transferred to 95% ethanol, followed by 100% ethanol and xylene dehydration before imaging. The slides were mounted with neutral balsam (C1795, Sigma) and allowed to air dry. Finally, imaging was performed using a microscope (Qu et al., 2023).

### 2.18 Western blot analysis

Protein lysates were prepared using RIPA lysis buffer containing PMSF (P0013B, Beyotime, Shanghai) and quantified using the BCA Protein Assay Kit (23225, Thermo Fisher Scientific, Rockford, IL, United States). Fifty micrograms of protein were mixed with 2× SDS sample buffer and boiled at 100°C for 5 min. Samples were then subjected to SDS-PAGE and transferred to PVDF membranes using the wet transfer method. The membranes were blocked with 5% non-fat milk at room temperature for 1 h and incubated overnight at 4°C with diluted primary antibodies, The primary antibodies used were: Anti-BMP4 (ab235114, 1:5,000, Abcam, United Kingdom), Anti-MBP (78896T, 1:1,000, Cell Signaling Technology), Anti-MOG (ab233549, 1:1,000, Abcam, United Kingdom), Anti-CCL5 (ab7198, 1:1,000, Abcam, United Kingdom), and Anti-β-Actin (ab8227, 1:1,000, Abcam, United Kingdom). The membrane was washed three times with TBST (91414, Sigma-Aldrich, Germany), each wash lasting 10 min, then incubated with HRP-conjugated secondary antibody, Goat Anti-Rabbit IgG H&L (HRP) (ab97051, 1:2000, Abcam, Cambridge, United Kingdom) for 1 h. After additional washing with TBST, the membrane was placed on a clean glass plate. Equal volumes of Solution A and Solution B from the Pierce™ ECL Detection Kit (Cat. No. 32209, Thermo Fisher Scientific) were mixed in the darkroom and applied to the membrane. The signal was visualized using the Bio-Rad Imaging System (ChemiDoc™ XRS+, BIO-RAD).

### 2.19 RT-qPCR

Total RNA was extracted from cells using TRIzol (15596026, Invitrogen, Carlsbad, CA, United States) and assessed for concentration and purity with the NanoDrop 2000 spectrophotometer (1011U, NanoDrop, United States). According to the manufacturer’s instructions, mRNA was reverse-transcribed into cDNA using the PrimeScript RT Reagent Kit (RR047A, Takara, Japan). Real-time fluorescence qPCR was performed on the ABI7500 system (7,500, ABI, United States) under the following conditions: initial denaturation at 95°C for 10 min, followed by 40 cycles of denaturation at 95°C for 10 s, annealing at 60°C for 20 s, and extension at 72°C for 34 s, using actin as an internal control. Relative gene expression levels were calculated using the 2^−ΔΔCT^ method: ΔΔCT = ΔCT_experiment_ - ΔCT_control_, where ΔCT = CT (target gene) - CT (internal reference), and relative expression = 2^−ΔΔCT^. Experiments were performed in triplicate, and primers were synthesized by TaKaRa (Table 1).

**TABLE 1:** Primer sequences.

Gene name	5’→3′ (Forward)	5’→3′ (Reverse)
β-actin (rat)	GTTGTCGACGACGAGCG	GCACAGAGCCTCGCCTT
MOP (rat)	GAG​GTT​CTC​GGA​TGA​AGG​AG	CAG​GGT​TGA​TCC​AGT​AGA​AGG
MBP (rat)	CAC​AGA​AGA​GAC​CCT​CAC​AGC​GAC	CCG​CTA​AAG​AAG​CGC​CCG​ATG​GA
IL-6 (rat)	TCC​TAC​CCC​AAC​TTC​CAA​TGC​TC	TTG​GAT​GGT​CTT​GGT​CCT​TAG​CC
IL-1B (rat)	CAC​CTC​TCA​AGC​AGA​GCA​CAG	GGG​TTC​CAT​GGT​GAA​GTC​AAC
TNF-a (rat)	AAA​TGG​GCT​CCC​TCT​CAT​CAG​TTC	TCT​GCT​TGG​TGG​TTT​GCT​ACG​AC

### 2.20 Enzyme-linked immunosorbent assay (ELISA) biochemical testing

Culture supernatants were centrifuged at 1,500 × g for 15 min. According to the ELISA kit instructions for IL-1β (rat, E-EL-R0012, Elabscience), TNF-α (rat, E-EL-R2856, Elabscience), and IL-6 (rat, E-EL-R0015, Elabscience), 100 μL of dilution buffer, 100 μL of sample, and 100 μL of standard were added to each well and incubated at 37°C for 90 min. Subsequently, 100 μL of biotinylated detection antibody was added to each well and incubated at 37°C for 1 h; the wells were then washed three times for 2 min each. After adding 100 μL of horseradish peroxidase-conjugate, the plates were incubated for 30 min at 37°C. Following five washes, the substrate reagent was added, and the plates were incubated in the dark at 37°C for 15 min before stopping the reaction. The absorbance was measured at 450 nm using an Epoch microplate spectrophotometer (Bio-Tek, Winooski, VT, United States). Each sample was assayed in triplicate (Zhang et al., 2022).

### 2.21 Immunofluorescence staining

OPCs were fixed in 4% paraformaldehyde (P0099, abclonal) for 15 min, followed by three washes with PBS. Cell membranes were permeabilized with 0.5% Triton X-100 in PBS (P0096, Beyotime) for 15 min at room temperature. Non-specific binding sites were blocked with 5% bovine serum albumin (ST2254, Beyotime) in PBS for 1 h, followed by three washes of 5 min each. Primary antibodies were applied against Anti-MOG (45268T, 1:200, Cell Signaling Technology) and Anti-MBP (78896T, 1:50, Cell Signaling Technology). After washing, the samples were incubated at room temperature for 1 h with the secondary antibody, Alexa Fluor 488 Goat Anti-Rabbit IgG (ab150077, Abcam), in the dark. DNA was counterstained with DAPI (RM02978, abclonal) (Zhao et al., 2025).

### 2.22 Cell apoptosis detection

After incubating OPCs at 37°C and 5% CO_2_ for 48 h, cells were cultured for 24 h following treatment with nanobundles. Cells were then collected and centrifuged in pre-chilled PBS at 1,000 × g for 5 min, the supernatant was discarded, and the process was repeated twice. Cells were resuspended in 500 μL of pre-chilled PBS and analyzed for apoptosis using the Annexin V-FITC/Propidium Iodide (PI) Double Staining Kit (C1062S, Beyotime) through flow cytometry (CytoFLEX, Beckman Coulter, Brea, CA, United States), with results analyzed using FlowJo software (Qian et al., 2024).

### 2.23 Construction and grouping of the TN rat model

The TN model was established using a chronic infraorbital nerve (ION) compression injury (ION-CCI) method. Rats were anesthetized by intraperitoneal injection of pentobarbital (40 mg/kg). A 0.5 cm incision was made on the facial skin between the left eye and the whisker pad, exposing the distal portion of the ION. Two loose knots were tied with 4–0 chromic gut sutures on the distal branches of the ION (separated by 2 mm). The facial skin was then sutured using 4–0 polyester thread. Rats in the sham surgery group underwent the same procedure, including the skin incision and dissection of the ION, but no nerve ligation was performed (Chen et al., 2023). Three days after surgery, rats were administered intravenous injections of 200 µL of nanomaterial at a dose equivalent to 5 mg/kg DMH1, or 3 mg/kg DMH1 every 4 days, starting from Day 0 (with injections on Days 0, 4, 8, and 12). To investigate the effect of CCL5 on OPCs in the LPS + NM@DMH1 group, recombinant CCL5 protein was administered via subcutaneous injection at a dose of 1.5 μg/kg body weight, twice daily for 2 weeks (Kim et al., 2015). The experimental groups were as follows: Sham group (no nerve ligation), Model group (ION chronic compression injury), Model + DMH1 group (ION chronic compression injury with intravenous DMH1 treatment), Model + NM@DMH1 group (ION chronic compression injury with NM treatment), Model + NM@DMH1 group (ION chronic compression injury with NM@DMH1 treatment), and Model + NM@DMH1+CCL5 group (ION chronic compression injury, NM@DMH1 treatment, and recombinant CCL5 protein injection). On Day 20, rats’ pain sensitivity was assessed, and they were subsequently sacrificed for tissue collection. Demyelinated tissues were analyzed by Western Blot, RT-qPCR, H&E staining, immunohistochemistry, and ELISA.

### 2.24 Assessment of mechanical pain hypersensitivity in rats

Von Frey filaments (Stoelting, IL, United States) were used to test the mechanical withdrawal threshold (MWT) in rats, and the experiment was conducted by a researcher who was blinded to the experimental groups. Before the Von Frey test, the animals were first acclimated to the testing apparatus for 1 week. The Von Frey filaments used in the experiment included 0.6, 1.0, 1.4, 2.0, 4.0, 6.0, 8.0, 10.0, and 15.0 g. MWT was measured from pre-operation to 28 days post-operation. During the experiment, the animals were individually placed in cages and allowed to adapt to the new environment for 30 min. The Von Frey filaments were applied to the infraorbital nerve (ION) region, specifically at the center of the facial whisker pad. The following behaviors were considered positive withdrawal responses: brisk head withdrawal, escape or attack reaction, and brief facial grooming. The up-down method was used to determine the 50% MWT, which represents the force at which the animal exhibits a withdrawal response with a 50% probability. Mechanical allodynia was recorded on days 0, 1, 4, 7, 14, 21, and 28 post-operation (Chen et al., 2023).

### 2.25 H&E staining

After euthanasia, samples of demyelinated trigeminal nerve tissue were collected, fixed in 4% formaldehyde solution, and embedded in paraffin. Tissue sections of 4 µm were cut and stained with H&E staining reagent (C0105M, Beyotime) for 1 minute, then rinsed with tap water until clear. Sections were counterstained with eosin for 15 s, followed immediately by dehydration in 95% ethanol and dehydration in 100% ethanol and xylene. Before imaging, sections were mounted using a neutral balsam fixative (C1795, Sigma) and air-dried. Microscopic imaging was performed. Inflammation was quantified on a scale of 0–5. The scoring was as follows: 0 = no inflammation, 1 = mild inflammation, 2 = mild/moderate inflammation, 3 = moderate inflammation, 4 = moderate/severe inflammation, and 5 = severe inflammation (Wang et al., 2024).

### 2.26 Immunohistochemistry (IHC)

Collected tissue samples were fixed in 4% formaldehyde solution (P0147A, Beyotime), embedded in paraffin, and sectioned into 4 μm slices. Antigen retrieval was performed by heating the sections in EDTA (P0085, Beyotime) in a microwave oven. The sections were treated with 3% hydrogen peroxide and blocked with goat serum (C0265, Beyotime). Primary antibodies were applied and incubated overnight at 4°C. The primary antibodies used in this study were Anti-BMP4 (ab235114, 1:200, Abcam, United Kingdom), Anti-MBP (78896T, 1:1,200, Cell Signaling Technology), and Anti-MOG (ab233549, 1:500, Abcam, United Kingdom). Sections were then incubated with biotinylated IgG secondary antibody (33103ES60, 1:100, Yeasen, China) followed by a 20-min incubation with streptavidin peroxidase reagent. Signal detection was completed using DAB reagent (P0202, Beyotime). Slides were scanned and analyzed using a Pannoramic Midi scanner (3DHISTECH) (Xia et al., 2020).

### 2.27 Statistical analysis

All data were derived from at least three independent experiments and are expressed as mean ± standard deviation (Mean ± SD). The Shapiro–Wilk test was used to assess data normality. For comparisons between two groups with normally distributed data and equal variance, two-tailed unpaired Student’s t-tests were applied. For comparisons among three or more groups, one-way analysis of variance (ANOVA) followed by Tukey’s honestly significant difference (HSD) *post hoc* test was used. If normality or homogeneity of variance was not met, non-parametric tests were applied: Mann–Whitney U test for two groups and Kruskal–Wallis H test for multiple groups, followed by Dunn’s test for pairwise comparisons if significant.

Two-way ANOVA was employed for experiments involving cellular viability at different time points to evaluate the main effects of time, treatment, and their interaction. For *in vivo* experiments involving repeated measurements across time points, a two-way repeated measures ANOVA was conducted to assess the effects of treatment, time, and their interaction.

All statistical analyses were performed using GraphPad Prism 9 (GraphPad Software, Inc.) and R software. A *p*-value <0.05 (two-tailed) was considered statistically significant. Detailed statistical outcomes, including p-values, degrees of freedom (df), and corresponding t or F values, are provided in the supplementary materials.

## 3 Results

### 3.1 Peptide nanobundles exhibit stability with uniform particle size and high thermal stability

Advancements in drug development for TN have been significant, yet limitations in delivery methods often reduce drug efficacy. Common drug delivery systems include liposomes, nanoparticles, and polymeric carriers. However, these methods face challenges such as poor *in vivo* stability, inadequate targeting, and potential side effects. Scientists have developed a novel drug-delivery material known as peptide nanobundles to address these issues. This material encapsulates hydrophobic drugs within its core, significantly enhancing the solubility of the drugs in water and thereby improving their bioavailability *in vivo*. Furthermore, peptide nanobundles offer excellent drug stability and biocompatibility, can target specific sites, and simplify the drug packaging process, making them a highly advantageous delivery method (Bose et al., 2021; Tawfik et al., 2020).

**FIGURE 1:**
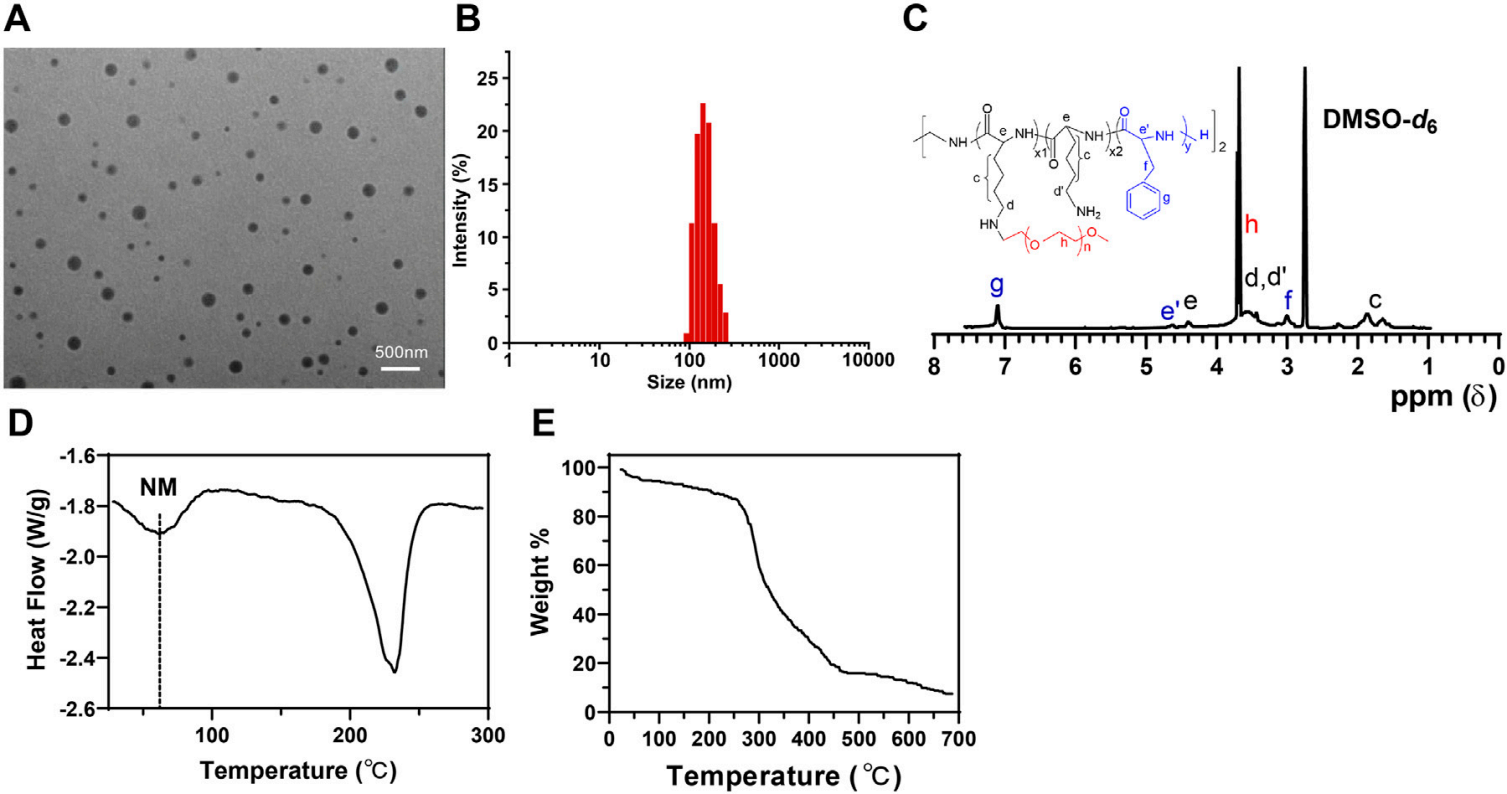
Characterization of peptide nanobundles. Note: **(A)** Structure of nanobundles observed with cryo-electron microscopy (scale: 500 nm); **(B)** Hydrodynamic diameter of nanobundles measured using DLS; **(C)** NMR spectrum of the amphiphilic copolymer mPEG-g-PLL-b-Phe measured using NMR; **(D)** Thermal transitions of nanobundles detected by DSC; **(E)** Thermal stability analysis of nanobundles conducted with TGA.

Our research focuses on peptide nanobundles to explore an ideal drug delivery model. Using self-assembly techniques, we have successfully prepared amphiphilic peptide nanobundles containing hydrophobic and hydrophilic parts. TEM images show that the nanobundles have a regular spherical structure with an average diameter of approximately 120 nm (Figure 1A). DLS results indicate that the particle size distribution ranges from 100–150 nm, demonstrating good particle size control and uniformity (Figure 1B). NMR spectra reveal chemical shifts at 7.11 ppm, 4.12 ppm, and 2.92 ppm corresponding to the phenyl ring (Hg), backbone (He’), and methylene (Hf) protons of the phenylalanine repeat units, confirming the successful synthesis of PLL-b-Phe. The peak at 3.52 ppm attributed to the ethylene protons of mPEG indicates the successful grafting of mPEG-CHO onto PLL-b-Phe, forming mPEG-g-PLL-b-Phe, thus confirming the successful synthesis of the amphiphilic mPEG-g-PLL-b-Phe (Figure 1C). DSC results show no significant thermal transitions before heating to 200°C, indicating high thermal stability (Figure 1D). TGA shows that peptide nanobundles degrade slowly between 300°C and 500°C, demonstrating good stability (Figure 1E), which confirms the uniform particle size and high thermal stability of peptide nanobundles.

### 3.2 Efficient loading and sustained release of DMH1 by peptide nanobundles

**FIGURE 2:**
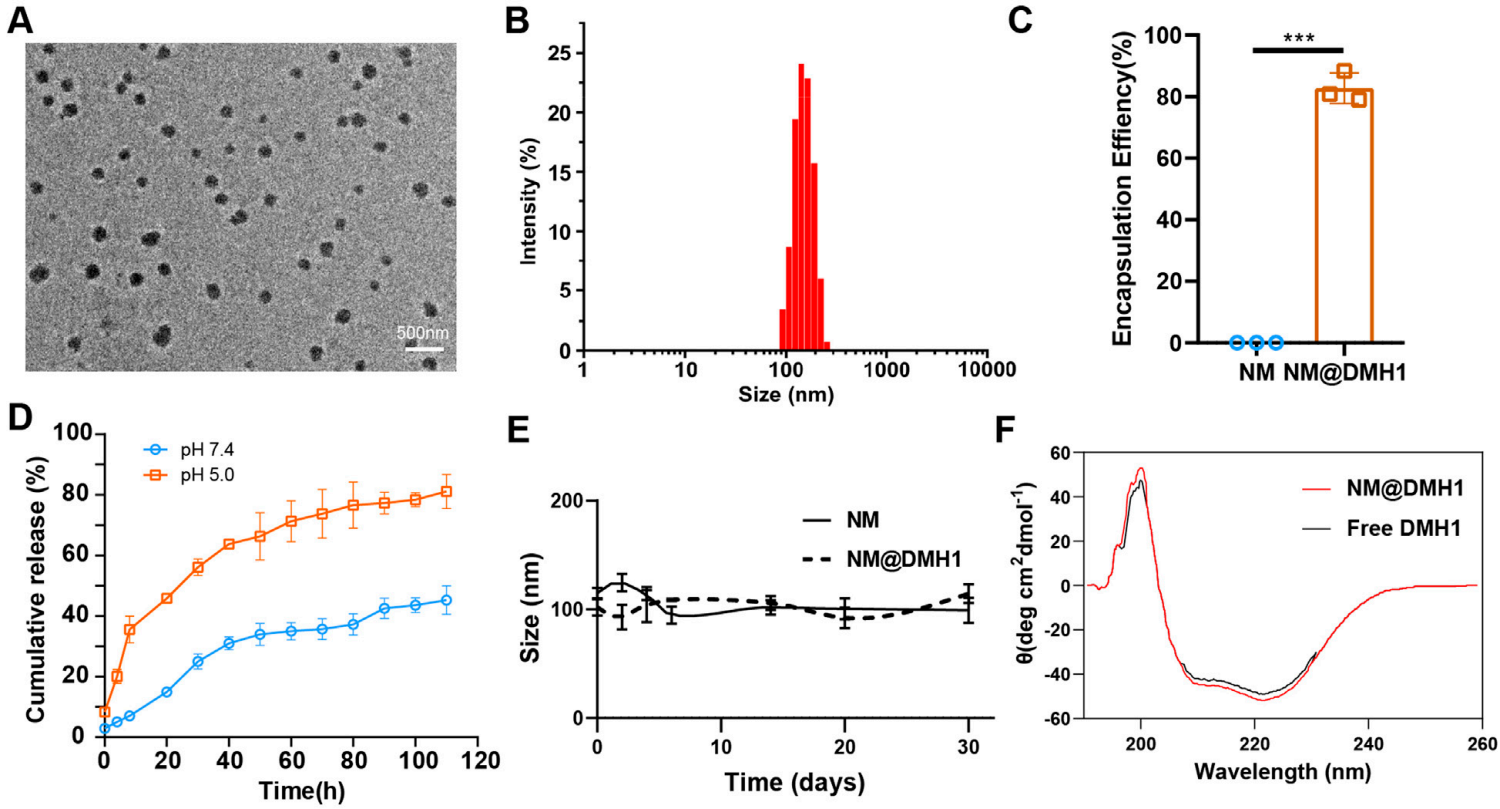
Characterization of DMH1-loaded peptide nanobundles. Note: **(A)** Structure of DMH1-loaded nanobundles observed with cryo-electron microscopy (scale: 500 nm); **(B)** Hydrodynamic diameter of DMH1-loaded nanobundles measured using DLS; **(C)** EE of NM@DMH1 and NM determined by ultracentrifugation; **(D)** Analysis of DMH1 release under different pH conditions using dialysis bag diffusion method; **(E)** Long-term storage stability of NM@DMH1 at 4°C tested; **(F)** Secondary structure of DMH1 analyzed by CD spectroscopy. Data are presented as mean ± SD (n = 3; ***p < 0.001).

Targeting the BMP4 signaling pathway offers a promising therapeutic strategy for TN, potentially serving as an effective intervention for disease progression. However, proteins without special treatment are challenging to deliver to critical sites via intravenous injection (Ma et al., 2024). To address this, we employed nanobundles to encapsulate BMP4 inhibitor DMH1 to enhance its therapeutic efficacy. TEM images reveal that the DMH1-loaded nanobundles maintained a regular spherical structure (Figure 2A). DLS results indicate a uniform particle size distribution between 100 and 150 nm, demonstrating that loading DMH1 does not compromise uniformity (Figure 2B). EE of NM@DMH1, indirectly measured using centrifugal ultrafiltration, reached 82% (Figure 2C). In release performance tests, at pH 7.4, DMH1 encapsulated within the nanomicelles released 45.3% of its content over 110 h; at pH 5.0, the release profile showed an initial burst followed by sustained release, with a release rate of 81.2% during the same period (Figure 2D). This indicates that NM@DMH1 exhibits stable, sustained-release, and pH-responsive release properties, with accelerated release under acidic conditions, ensuring effective drug delivery to specific inflammatory sites. Stability tests reveal little change in the particle size of the various nanomicelles after 30 days of storage at 4°C ± 2°C (Figure 2E). CD analysis indicated that the secondary structure of DMH1 within the nanobundles remained stable before and after loading, with no significant changes (Figure 2F). These results demonstrate that peptide nanobundles can efficiently load DMH1 and maintain its stability, providing important evidence for its clinical application.

### 3.3 Efficient uptake and good compatibility of peptide Nanobundles@DMH1 by OPCs

**FIGURE 3:**
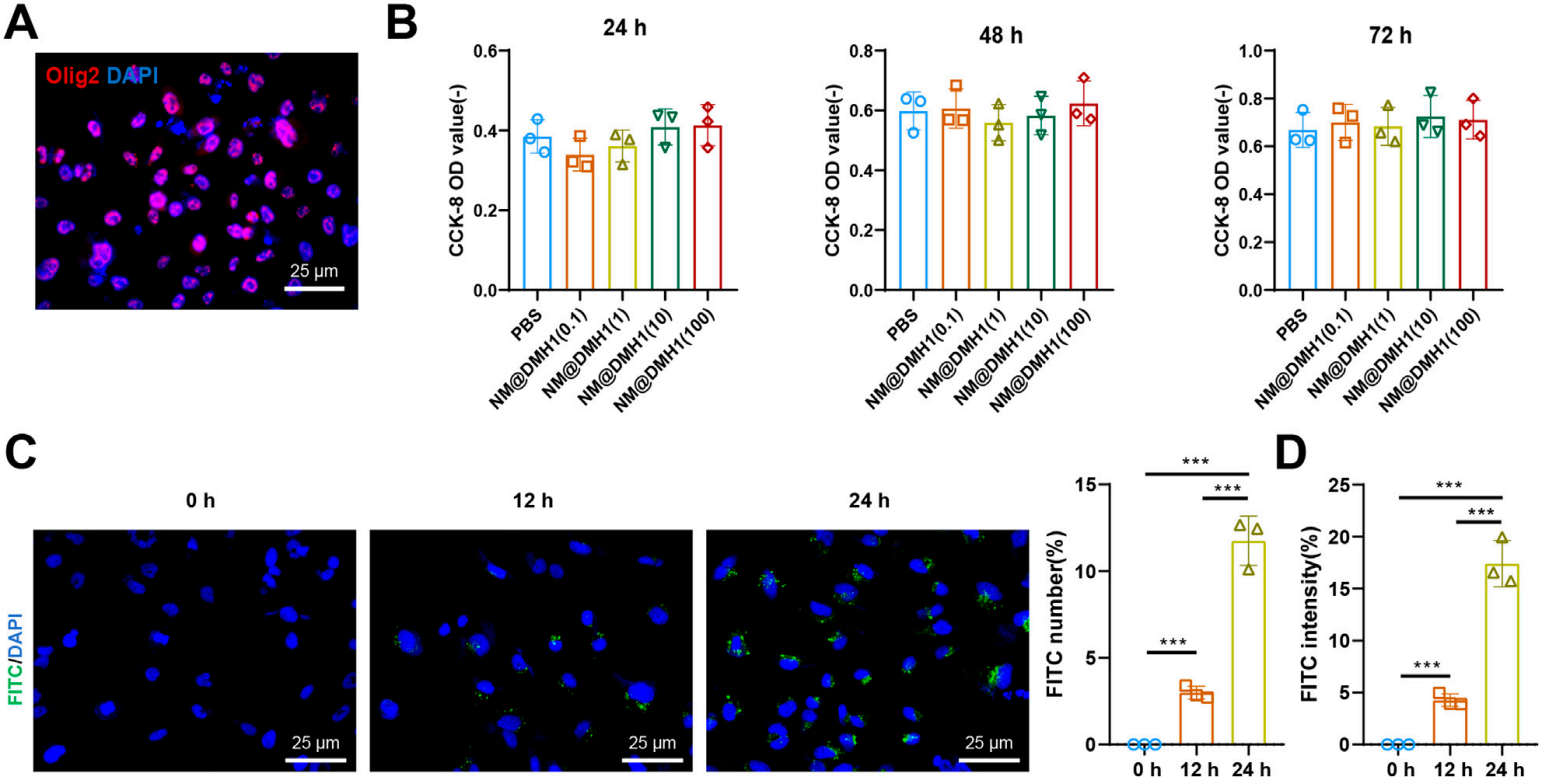
Cellular uptake and biocompatibility of DMH1-loaded nanobundles. Note: **(A)** Immunofluorescence detection of Oligs expression in OPCs (scale: 25 µm); **(B)** Cell toxicity of NM@DMH1 on OPCs tested using CCK-8 assay; **(C)** Immunofluorescence detection of NM@DMH1 uptake by OPCs (scale: 25 µm); **(D)** Flow cytometry analysis of immunofluorescence detected uptake of NM@DMH1 by OPCs (***p < 0.001, experiments repeated three times).

**FIGURE 4:**
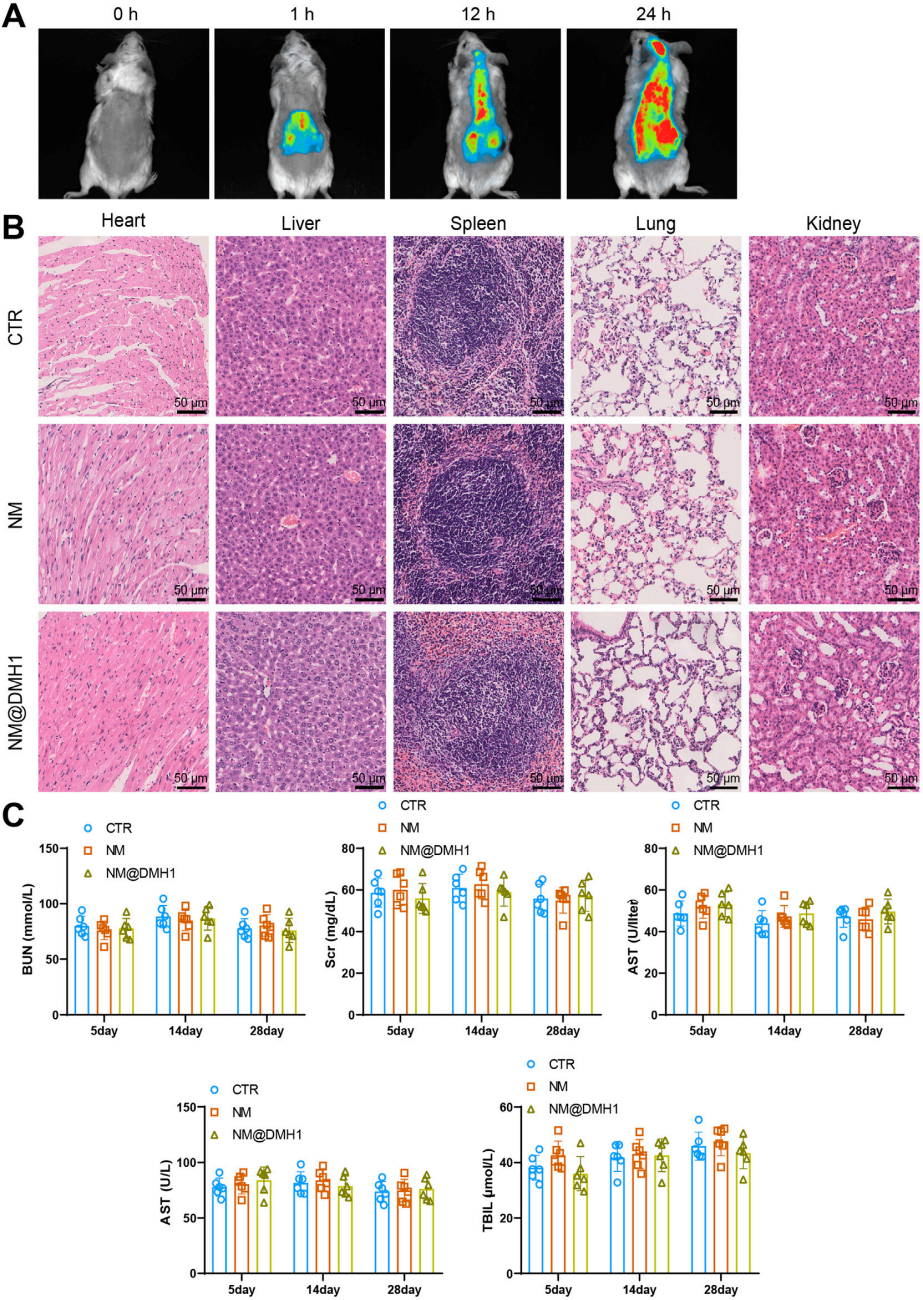
*In vivo* distribution and biosafety evaluation of NM@DMH1. Note: **(A)** IVIS tracking the distribution of NM@DMH1 in rats; **(B)** H&E staining to assess the toxicity in the heart, liver, spleen, lungs, and kidneys after 28 days of NM@DMH1 injection (scale: 50 µm); **(C)** Biochemical analysis of whole blood samples from rats on days 5, 14, and 28 post NM@DMH1 injection. Data are presented as mean ± SD (n = 6).

OPCs were successfully isolated from neonatal rat brain tissue and identified by immunofluorescence staining. The results show positive staining for the OPC marker Olig2 (Figure 3A). Additionally, the cytotoxic effects of NM@DMH1 on OPCs were assessed using the CCK-8 assay kit. After treating OPCs with various concentrations for 24, 48, and 72 h, NM@DMH1 showed no significant impact on cell viability compared to the control group, indicating no apparent toxicity of NM@DMH1 to OPCs (Figure 3B). The uptake efficiency of FITC-labeled NM@DMH1 by OPCs was monitored using fluorescence microscopy and flow cytometry. The results demonstrated an increase in FITC fluorescence intensity over time, suggesting that the peptide nanobundles were efficiently internalized by the cells, and the uptake by OPCs increased time-dependent (Figures 3C,D). After intravenous injection of FITC-labeled peptide nanobundles@DMH1 into rats, their bodily distribution was tracked using an IVIS. Results indicate that 1 hour post-injection, the nanomicelles were primarily distributed in the liver and kidneys, with a gradual redistribution to the brain observed between 12 and 24 h (Figure 4A). Detailed biological evaluations were conducted on the intravenously injected nanobundles regarding biocompatibility assessment. After 28 days, H&E staining of organs such as the heart, liver, spleen, lungs, and kidneys showed no signs of inflammatory infiltration or pathological changes (Figure 4B). Further biochemical analysis of arterial blood samples collected on days 5, 14, and 28 post-injection showed that BUN, Scr, ALT, AST, and TBIL levels were within normal ranges, with no significant differences from the control group (Figure 4C). These results indicate that peptide nanobundles are efficiently taken up by OPCs and exhibit good biocompatibility.

### 3.4 Peptide Nanobundles@DMH1 induce differentiation of OPCs and suppress inflammation

Previous research indicates that OPCs can differentiate into mature oligodendrocytes capable of myelination, thus repairing demyelinating lesions in rats with TN (Skaper, 2019). However, the upregulation of pro-inflammatory cytokines inhibits OPC differentiation (Shao et al., 2021). Since DMH1 has been shown to promote OPC differentiation, this study explored whether NM@DMH1 could effectively facilitate OPC differentiation while reducing inflammation levels (Choe et al., 2014).

**FIGURE 5:**
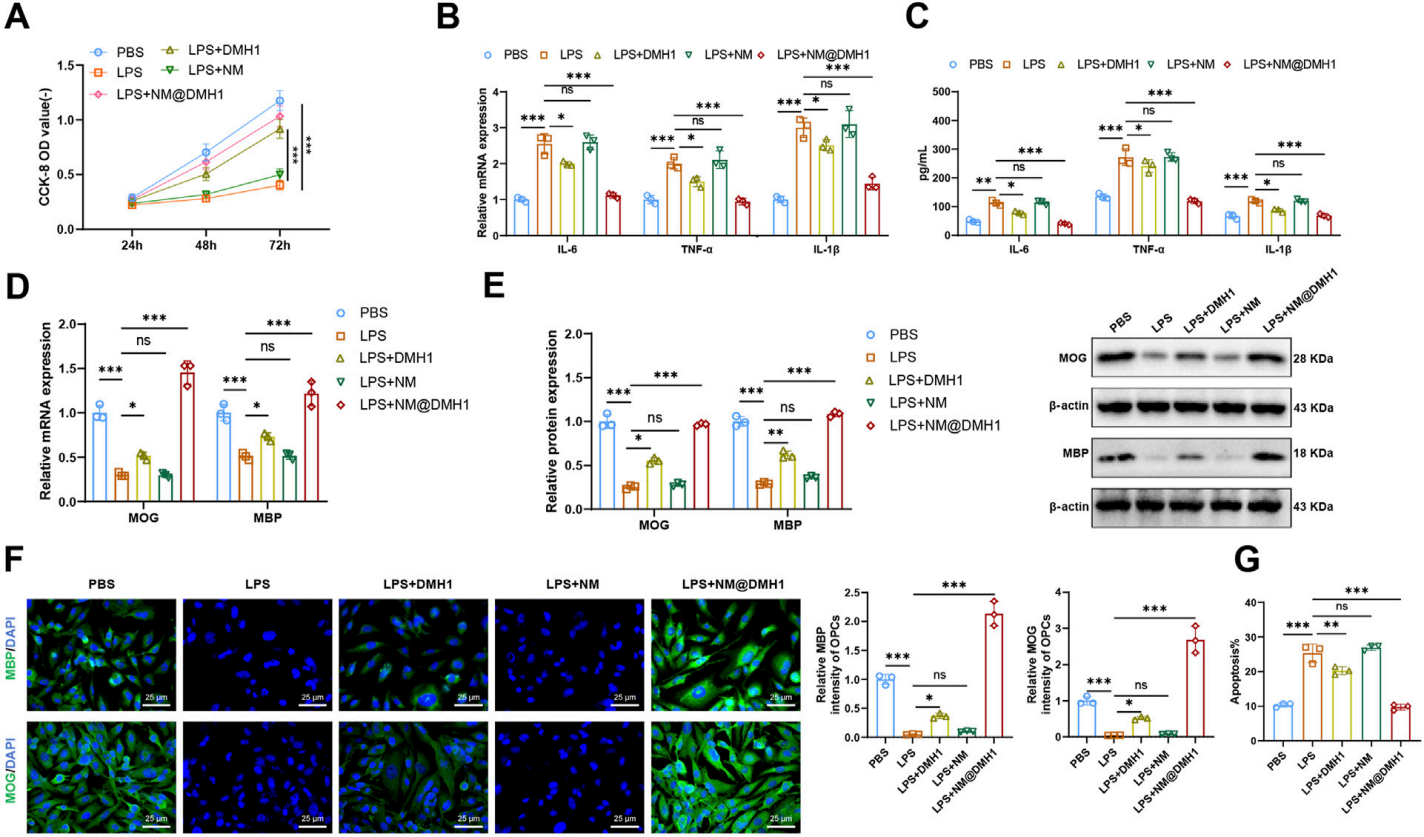
Effects of peptide nanobundles@DMH1 on differentiation and inflammation in OPCs. Note: **(A)** CCK-8 assay measuring OPC viability at 24, 48, and 72 h; **(B)** RT-qPCR analysis of pro-inflammatory cytokines in OPCs; **(C)** ELISA quantification of pro-inflammatory cytokines in OPCs; **(D)** RT-qPCR of OPC differentiation markers; **(E)** Western blot of OPC differentiation markers; **(F)** Immunofluorescence detection of OPC differentiation markers (scale bar: 25 μm); **(G)** Flow cytometry analysis of apoptosis in OPCs. In all cell-based experiments, LPS was applied at 15 μg/mL (200 µL), DMH1 at 20 μg/mL (200 µL), NM (vehicle micelles) at 200 μL, and NM@DMH1 at 20 μg/mL (200 µL). Data are presented as mean ± SD (ns: not significant, *p < 0.05, **p < 0.01, ***p < 0.001, experiments repeated three times).

To investigate the regulatory effects of NM@DMH1 on OPCs under inflammatory conditions, we constructed an inflammation model by treating cells with LPS and initially assessed cell viability using the CCK-8 assay (Figure 5A). Compared to the PBS group, the LPS group showed significantly reduced OPC viability, indicating cytotoxic effects of LPS. In contrast, the LPS + DMH1 and LPS + NM@DMH1 groups demonstrated significantly restored cell viability, with the LPS + NM@DMH1 group showing a greater recovery, approaching levels observed in the PBS group. There was no significant difference between the LPS and LPS + NM groups, suggesting that the drug-free nanocarrier had no detectable effect on cell viability. Additionally, we assessed the expression of IL-6, TNF-α, and IL-1β using RT-qPCR and ELISA (Figures 4B,C).

We observed that, compared to the PBS control group, the LPS group showed a significant upregulation of pro-inflammatory cytokines (IL-6, TNF-α, and IL-1β), indicating a highly inflammatory state in OPCs. There was no significant change in the expression levels of IL-6, TNF-α, and IL-1β in the LPS + NM group compared to the LPS group. However, these levels were significantly reduced in the LPS + DMH1 and LPS + NM@DMH1 groups, with the most notable reduction observed in the NM@DMH1 treatment group, suggesting effective mitigation of inflammation by NM@DMH1. Further analyses via RT-qPCR and Western Blot (Figures 5D,E) showed that the expression of the differentiation markers MBP and MOG was significantly decreased in the LPS group compared to the PBS controls. In contrast, the expressions of MBP and MOG were significantly upregulated in the LPS + DMH1 and LPS + NM@DMH1 groups, with NM@DMH1 treatment yielding the highest levels, indicating successful promotion of OPC differentiation by NM@DMH1. Immunofluorescence analysis revealed (Figure 5F) a significant downregulation of MBP and MOG fluorescence signals in OPCs of the LPS group compared to PBS controls. No significant changes were observed in the LPS + NM group, whereas the LPS + DMH1 and LPS + NM@DMH1 groups exhibited notably increased fluorescence signals for these markers, especially in the LPS + NM@DMH1 group. Flow cytometry analysis (Figure 5G) showed that the rate of cell apoptosis in the LPS + NM@DMH1 group was significantly lower than in the LPS group.

These results suggest that NM@DMH1 not only significantly promotes the differentiation of OPCs but also effectively suppresses the expression of inflammation-related factors and reduces the rate of cell apoptosis, demonstrating its potential application in treating related diseases.

### 3.5 NM@DMH1 in alleviating demyelination in a rat model of TN

**FIGURE 6:**
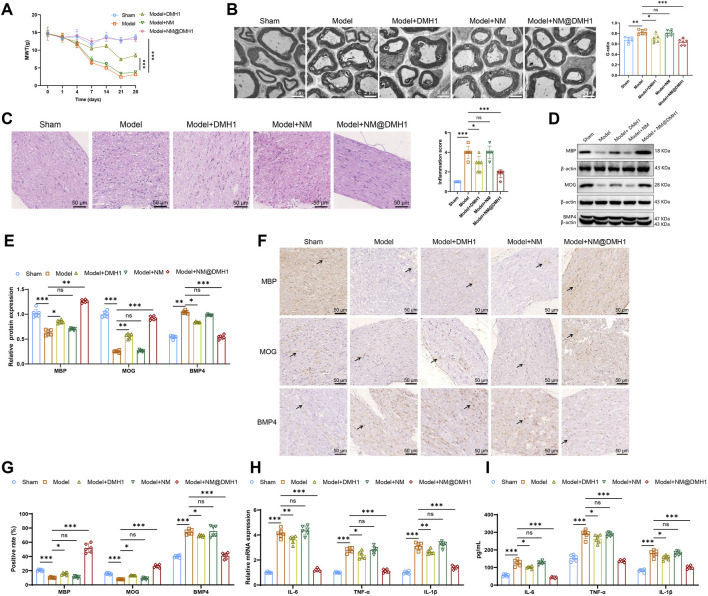
Treatment effects of peptide nanobundles@DMH1 on demyelinating lesions in rats with TN. Note: **(A)** Assessment of mechanical allodynia thresholds in rats (n = 6); **(B)** TEM observations of myelin structures in rats (scale: 2 μm) (n = 6); **(C)** H&E staining to assess inflammation in demyelinated trigeminal nerve tissues of TN model rats (scale: 50 μm) (n = 6); **(D,E)** Western Blot analysis of key differentiation proteins in OPCs (n = 6); **(F,G)** IHC analysis of key differentiation proteins in OPCs (scale: 50 μm) (n = 6); **(H)** RT-qPCR analysis of pro-inflammatory factor expression (n = 6); **(I)** ELISA for quantification of pro-inflammatory factors (n = 6). For *in vivo* studies, rats received tail vein injections of NM@DMH1 at a dose equivalent to 5 mg DMH1/kg in a volume of 200 µL. Data are presented as mean ± SD (ns: not significant, *p < 0.05, **p < 0.01, ***p < 0.001).

To further validate the therapeutic efficacy of NM@DMH1 *in vivo*, we established a TN model in SD rats. In tests for mechanical allodynia, the pain threshold of the Model group was lower than that of the Sham group. The Model + NM and Model + DMH1 groups showed no significant difference and a slight increase in pain threshold, respectively. However, the Model + NM@DMH1 group displayed a significant increase in pain threshold, indicating a reversal of hypersensitivity (Figure 6A). TEM of myelin structures (Figure 6B) revealed that while the Sham group showed intact and uniform myelin, the Model and Model + NM groups exhibited noticeable demyelination, characterized by sparse or fragmented myelin. The Model + DMH1 group showed some reduction in demyelination, but significant defects remained. In contrast, the Model + NM@DMH1 group showed markedly reduced demyelination with myelin thickness approaching normal levels. H&E staining confirmed these observations: the Model group displayed significant inflammatory cell infiltration and patchy demyelination, whereas the Model + NM@DMH1 group showed substantially reduced inflammation and more intact myelin structures (Figure 6C).

Western Blot analysis revealed that compared to the Sham group, the Model group had significantly decreased expression of MBP and MOG proteins and increased expression of BMP4. There were no significant changes in the Model + NM group, but in the Model + NM@DMH1 group, MBP and MOG levels were significantly higher, and BMP4 expression was reduced (Figures 6D,E). IHC results were consistent with these findings (Figures 6F,G). Additionally, RT-qPCR and ELISA analyses of inflammatory markers showed that, compared to the Sham group, the Model group had elevated levels of inflammatory factors. While the Model + DMH1 group showed a slight decrease, the Model + NM@DMH1 group exhibited a significant reduction in inflammatory factor expression (Figures 6H,I). These results indicate that NM@DMH1 effectively alleviates demyelination and reduces inflammation in a rat model of TN.

### 3.6 Reversal of NM@DMH1 effects on OPC differentiation and inflammation by CCL5

**FIGURE 7:**
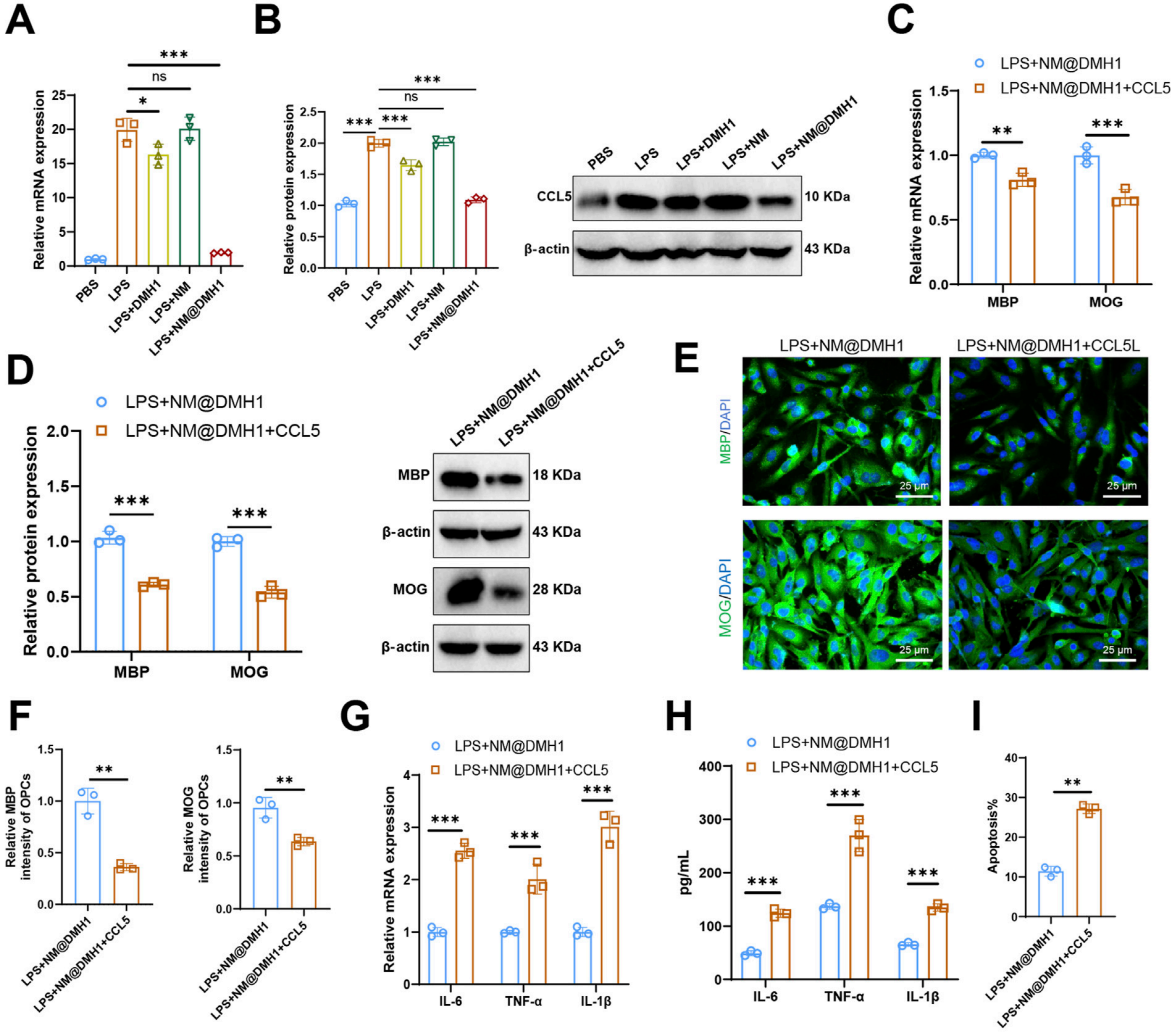
Regulation of CCL5 expression by DMH1 and its impact on OPC differentiation and inflammatory factors. Note: **(A)** RT-qPCR analysis of chemokine CCL5 mRNA expression levels in OPCs; **(B)** Western Blot analysis of chemokine CCL5 protein levels in OPCs; **(C)** RT-qPCR analysis of mRNA expression levels of differentiation markers MBP and MOG in OPCs; **(D)** Western Blot analysis of protein expression levels of differentiation markers MBP and MOG in OPCs; **(E,F)** Immunofluorescence detection of protein expression of differentiation markers MBP and MOG in OPCs (scale: 25 μm); **(G)** RT-qPCR analysis of mRNA expression levels of pro-inflammatory factors IL-6 and TNF-α; **(H)** ELISA for protein expression levels of pro-inflammatory factors IL-6 and TNF-α; **(I)** Flow cytometry analysis of apoptosis rates in OPCs. In cell experiments, the treatment dosages were as follows: LPS at 15 μg/mL (200 µL), DMH1 at 20 μg/mL (200 µL), NM at 200 μL, and NM@DMH1 at 20 μg/mL (200 µL). Data are presented as mean ± SD (**p < 0.01, ***p < 0.001, experiments repeated three times).

Research indicates that CCL5 plays a crucial role as a downstream pathway in the BMP signaling route in demyelinating diseases of the central nervous system, such as multiple sclerosis (Tang et al., 2022). We analyzed the expression levels of the chemokine CCL5 in different treatment groups using RT-qPCR and Western Blot. Results (Figures 7A,B) show that LPS treatment significantly upregulated CCL5 expression in OPCs compared to the PBS group. Further analysis revealed that CCL5 expression did not significantly change in the LPS + NM group but was significantly reduced in the LPS + DMH1 and LPS + NM@DMH1 groups, with the most substantial reduction in the LPS + NM@DMH1 group. To examine the role of CCL5 in peptide nanomicelle-facilitated OPC differentiation, we treated OPCs in the LPS + NM@DMH1 group with recombinant CCL5 protein. RT-qPCR and Western Blot analyses (Figures 7C,D) demonstrated that the addition of CCL5 significantly inhibited the expression of differentiation markers MBP and MOG compared to the LPS + NM@DMH1 group. Immunofluorescence assays further confirmed that the expression of OPC differentiation markers significantly decreased after treatment with recombinant CCL5 (Figures 7E,F). Additionally, RT-qPCR and ELISA results (Figures 7G,H) showed that the addition of CCL5 significantly increased the expression of pro-inflammatory cytokines IL-6 and TNF-α. Flow cytometry analysis revealed that the apoptosis rate in OPCs of the LPS + NM@DMH1 group significantly increased after adding CCL5 (Figure 7I). These findings suggest that CCL5 can reverse the protective effects of NM@DMH1 by inhibiting differentiation and exacerbating inflammation.

### 3.7 NM@DMH1 mitigates demyelination in a TN rat model by suppressing CCL5 expression and reducing immune cell infiltration

**FIGURE 8:**
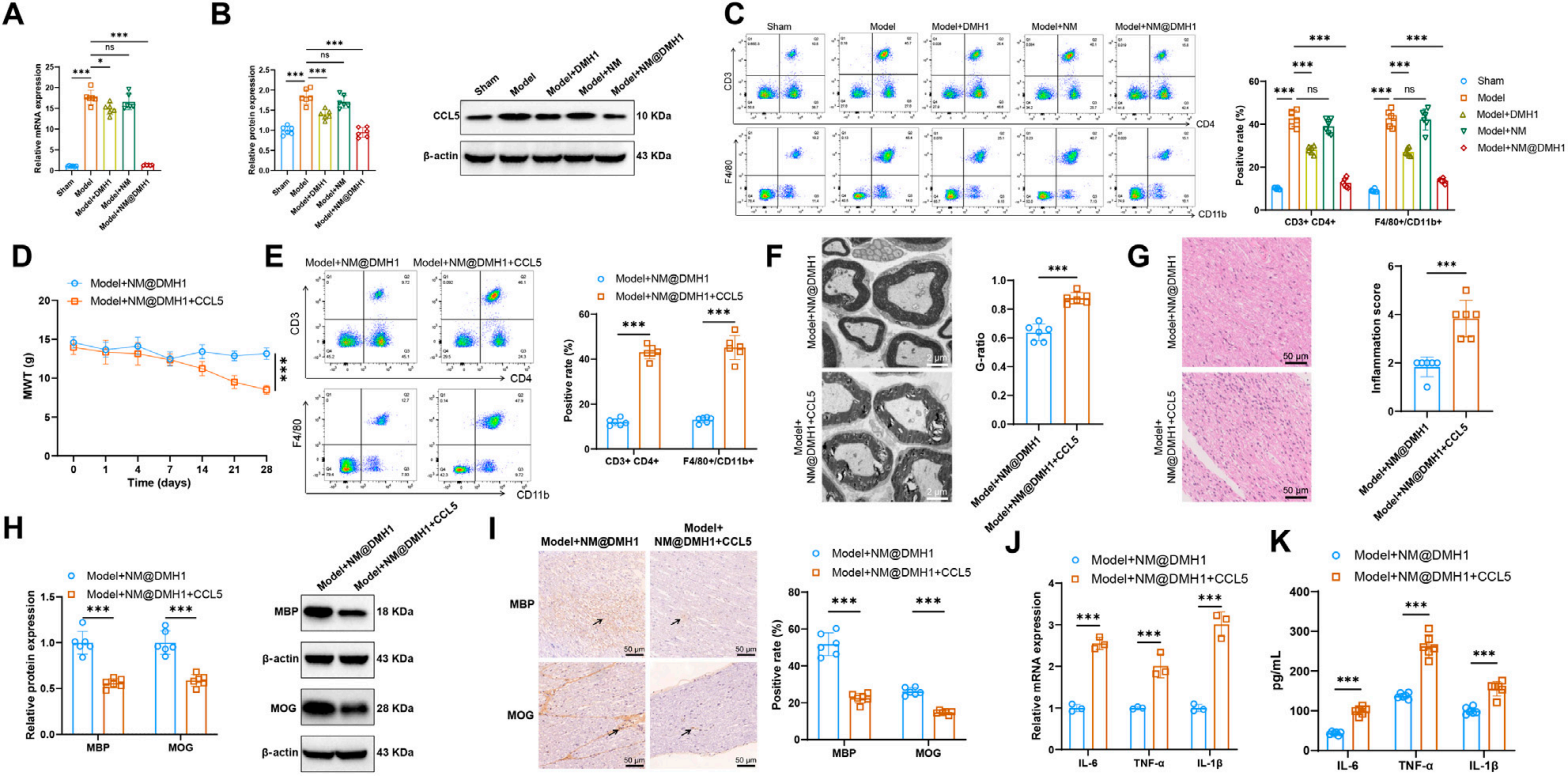
Regulatory effects of NM@DMH1 on CCL5 expression and inflammatory responses in a rat model of TN. Note: **(A)** RT-qPCR analysis of CCL5 mRNA levels in trigeminal nerve tissues; **(B)** Western Blot analysis of CCL5 protein levels in trigeminal nerve tissues; **(C)** Flow cytometry measurement of infiltrating T cells (CD3^+^CD4^+^) and macrophages (F4/80^+^/CD11b^+^) in rat brain tissue; **(D)** Mechanical allodynia test to assess mechanical pain thresholds in rats; **(E)** Flow cytometry analysis of the proportion of infiltrating T cells (CD3/CD4) and macrophages (F4/80^+^/CD11b^+^) in rat brain tissue; **(F)** TEM to observe the integrity of the myelin structure in the trigeminal nerve; **(G)** H&E staining to examine inflammatory cell infiltration in trigeminal nerve tissues; **(H)** Western Blot analysis of MBP, MOG, and BMP4 protein expression in trigeminal nerve tissues; **(I)** IHC analysis of MBP, MOG, and BMP4 protein expression in trigeminal nerve tissues; **(J)** RT-qPCR analysis of pro-inflammatory factor expression in trigeminal nerve tissues; **(K)** ELISA to quantify pro-inflammatory factor levels in trigeminal nerve tissues. Data are presented as mean ± SD (*p < 0.05, ***p < 0.001, n = 6).

RT-qPCR and Western Blot analyses revealed that, compared to the Sham group, the expression levels of CCL5 mRNA and protein were significantly elevated in both the Model group and the Model + NM group. In the Model + DMH1 group, CCL5 expression decreased compared to the Model group, but did not return to baseline levels; however, in the Model + NM@DMH1 group, CCL5 expression was significantly lower than in the Model group and approached levels seen in the Sham group (Figures 8A,B). Flow cytometry analysis showed a significant increase in infiltrating T cells (CD3^+^CD4^+^) and macrophages (F4/80^+^/CD11b^+^) in the brain tissue of the Model group and Model + NM group compared to the Sham group. This infiltration was reduced in the Model + DMH1 group, and significantly lower in the Model + NM@DMH1 group, nearing Sham group levels (Figure 8C). These findings suggest that NM@DMH1 significantly inhibits the aberrant expression of CCL5 in the TN model, potentially alleviating the inflammatory response through this pathway.

To further investigate the impact of CCL5 on the therapeutic effects of NM@DMH1, rats in the Model + NM@DMH1 group were treated with intraperitoneal injections of recombinant CCL5. Mechanical allodynia tests indicated that, compared to the Model + NM@DMH1 group, the Model + NM@DMH1+CCL5 group exhibited significantly decreased mechanical pain thresholds (Figure 8D). Flow cytometry analysis revealed a significant increase in infiltrating T cells and macrophages in the brain tissue of the Model + NM@DMH1+CCL5 group (Figure 8E). Electron microscopy and H&E staining confirmed that the Model + NM@DMH1+CCL5 group showed significantly worsened trigeminal nerve demyelination and increased inflammatory cell infiltration (Figures 8F,G). Western Blot and IHC results showed that the addition of CCL5 significantly reduced the expression levels of MBP and MOG proteins compared to the Model + NM@DMH1 group (*p* < 0.01) (Figures 8H,I). RT-qPCR and ELISA further confirmed that the expression levels of pro-inflammatory cytokines IL-6, TNF-α, and IL-1β were significantly elevated following the addition of CCL5 (Figures 8J,K). These results demonstrate that exogenous CCL5 promotes immune cell infiltration and can reverse the protective effects of NM@DMH1 against demyelination and inflammatory responses in TN.

## 4 Discussion

TN is a chronic pain condition that severely affects patients’ quality of life, characterized by sudden, intense electric shock-like pains primarily affecting the facial region (Lambru et al., 2021). It is characterized by sudden, intense facial pain, typically localized to the areas covered by the affected nerve branches. The severity and abrupt onset of the pain can cause significant distress in daily life (Araya et al., 2020). Current treatment options like pharmacotherapy and nerve blocking provide only short-term relief and often come with side effects, not effectively addressing the needs of all patients, particularly those unresponsive to medications (McMillan, 2011). Against this backdrop, our study explores the potential therapeutic effects of exogenous BMP4 inhibitor DMH1 delivered via peptide nanobundles on TN, especially its application in alleviating NP and demyelinating lesions.

This study utilizes nanotechnology, specifically peptide nanobundles, to optimize the delivery of BMP4 inhibitor DMH1. This nanoscale drug delivery system has displayed tremendous potential in enhancing drug bioavailability and targeting, offering new strategies and tools for treating neurological conditions such as TN (Kataria et al., 2024).

In this study, exogenous BMP4 inhibitor DMH1 significantly promoted the differentiation of OPCs, aligning with numerous findings in the neuroscience field. Previous research has demonstrated that BMP4 plays a crucial role in the activation of astrocytes during spinal cord injury, possibly initiating neuroglial activation through the p-Smad 1/5/8 and p-STAT3 signaling pathways, thereby exacerbating NP in rats (Yang et al., 2019). Given BMP4’s central role in these processes, targeting the BMP4 signaling pathway may offer new strategies and potential targets for the treatment of TN. However, distinct from previous research, our study leverages the advantages of nanotechnology by delivering BMP4 inhibitor DMH1 effectively through the peptide nanobundles system, enhancing its efficacy and targeting within specific neural environments, a topic seldom reported in previous literature.

The application of nanotechnology in the medical field is becoming increasingly widespread, particularly in drug delivery systems (Patra et al., 2018). Compared to traditional delivery systems such as liposomes and polymeric microspheres, peptide nanobundles offer enhanced biocompatibility and customizability (Wang and Chen, 2024). In this study, peptide nanobundles have been demonstrated to be an efficient carrier for BMP4 inhibitor DMH1, which is crucial for treating TN. By precisely controlling the size and surface properties of the nanobundles, we successfully enhanced the accumulation and sustained release of BMP4 inhibitor DMH1 in targeted tissues, which has often been challenging to achieve in previous studies. Furthermore, our approach reduces the risk of systemic side effects, showing better patient adaptability and potential for clinical application.

In this study, we utilized a rat model of TN and observed a significant reduction in pain sensitivity following treatment. These findings align with previous studies involving other neuroprotective agents or anti-inflammatory drugs. However, a distinctive aspect of our study is the notably enhanced improvement in demyelination lesions. This marked improvement can likely be attributed to the dual role of BMP4 inhibitor DMH1 in neuroprotection and promoting cell differentiation. Moreover, compared to commonly used medications in previous research, our treatment approach using peptide nanobundles@DMH1 demonstrated excellent safety, with no significant toxicity or adverse reactions observed. Ensuring treatment safety is crucial in the management of NP, making our findings a valuable reference for future therapeutic strategies.

In the context of neuropathic pain such as TN, accumulating evidence suggests that oligodendrocyte progenitor cells (OPCs) play not only a critical role in remyelination but also potentially contribute to the regulation of neuroinflammation (Zveik et al., 2024). In our study, we observed significant infiltration of CD4^+^ T cells and F4/80^+^CD11b^+^ macrophages in the brain tissues of model and treatment group rats, suggesting that OPCs may secrete chemokines to recruit immune cells, foster an inflammatory microenvironment, and thereby exacerbate demyelinating pathology. This phenomenon aligns with findings in recent studies of multiple sclerosis (MS) mouse models (Wang et al., 2025). In particular, using an EAE model with hyperactivation of the Wnt pathway, it was shown that OPCs can modulate chemokine expression to recruit CD4^+^ T cells and Gr-1^+^ cytotoxic macrophages, resulting in pronounced demyelination within the central nervous system.

CCL5 is a canonical pro-inflammatory chemokine that can be secreted by neurons, astrocytes, and OPCs in response to inflammatory stimuli (Pittaluga, 2017). In our study, exogenous administration of CCL5 reversed the protective effects of NM@DMH1 on demyelination and inflammation, further supporting its role as a key inflammatory mediator. Collectively, our results suggest that CCL5 contributes to the pathogenesis of TN by promoting immune cell infiltration and interfering with OPC differentiation.

While this study demonstrates the potential of peptide nanobundles in treating TN, their clinical application still faces significant challenges. For instance, the production costs and technical difficulties associated with scaling up are major barriers to widespread adoption. Additionally, this novel type of nanobundle requires validation in more extensive preclinical trials. Although the TN rat model used is widely accepted, it cannot fully replicate the complexity of human TN. Moreover, this study’s relatively small sample size may impact the statistical significance and generalizability of the results. Therefore, addressing these challenges will require future research to explore more cost-effective and efficient production methods and to further validate safety and efficacy in larger sample sizes, diverse animal models, and eventual clinical trials.

## Data Availability

The data presented in the study are deposited in the Figshare repository, DOI: https://doi.org/10.6084/m9.figshare.29595185.

## Glossary

ALT: Alanine Aminotransferase
ANOVA: Analysis of Variance
AST: Aspartate Aminotransferase
BUN: Blood Urea Nitrogen
BMP4: Bone Morphogenetic Protein 4
CCL5: C-C Motif Chemokine Ligand 5
CD: Circular Dichroism
DMH1: Dorsomorphin Homolog 1
DLS: Dynamic Light Scattering
DSC: Differential Scanning Calorimetry
EE: Encapsulation Efficiency
ELISA: Enzyme-Linked Immunosorbent Assay
H&E: Hematoxylin and Eosin
HPLC: High-Performance Liquid Chromatography
IHC: Immunohistochemistry
ION: Infraorbital Nerve
IVIS: *In Vivo* Imaging System
LPS: Lipopolysaccharide
MWCO: Molecular Weight Cutoff
NMR: Nuclear Magnetic Resonance
NP: Neuropathic Pain
OD: Optical Density
OPCs: Oligodendrocyte Progenitor Cells
PBS: Phosphate-Buffered Saline
PDI: Polydispersity Index
PI: Propidium Iodide
qPCR: Real-Time Quantitative PCR
ROP: Ring-Opening Polymerization
Scr: Serum Creatinine
SD: Sprague Dawley
TN: Trigeminal Neuralgia
TGA: Thermogravimetric Analysis
TGF-β: Transforming Growth Factor β
TBIL: Total Bilirubin
TEM: Transmission Electron Microscopy
