# Supplementary figures and images for: DMH1-loaded peptide nanomicelles restore myelin and attenuate neuroinflammation in trigeminal neuralgia via CCL5 suppression
Source: Front Pharmacol. 2025 Aug 6;16:1590624. doi: 10.3389/fphar.2025.1590624 (PMC12364832; doi:10.3389/fphar.2025.1590624)

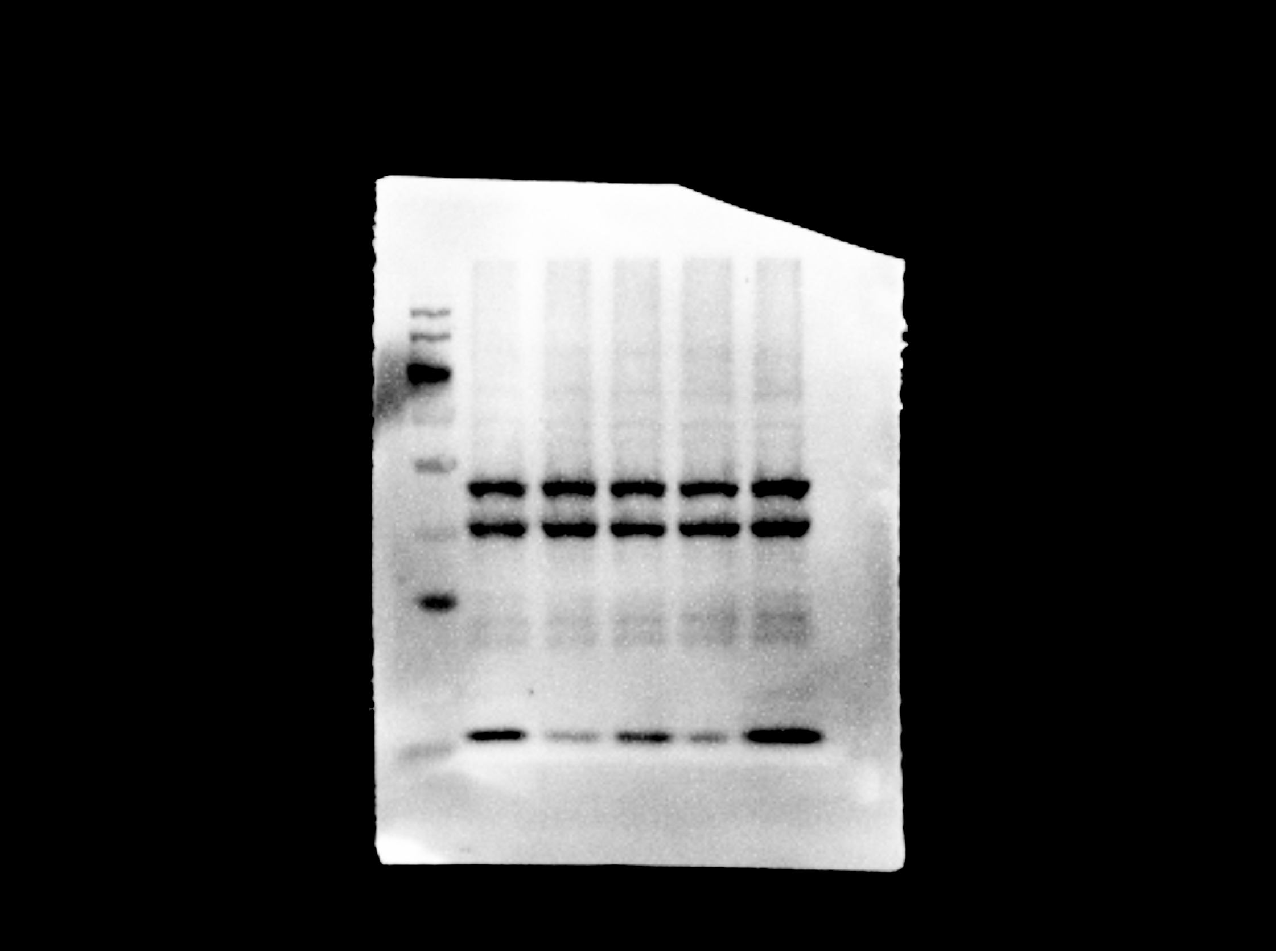

Supplement: Supplementary file 1 [file DataSheet3.ZIP › Full and uncropped western blots of Figure 6/Full and uncropped western blots of Figure 6D-1.jpg]

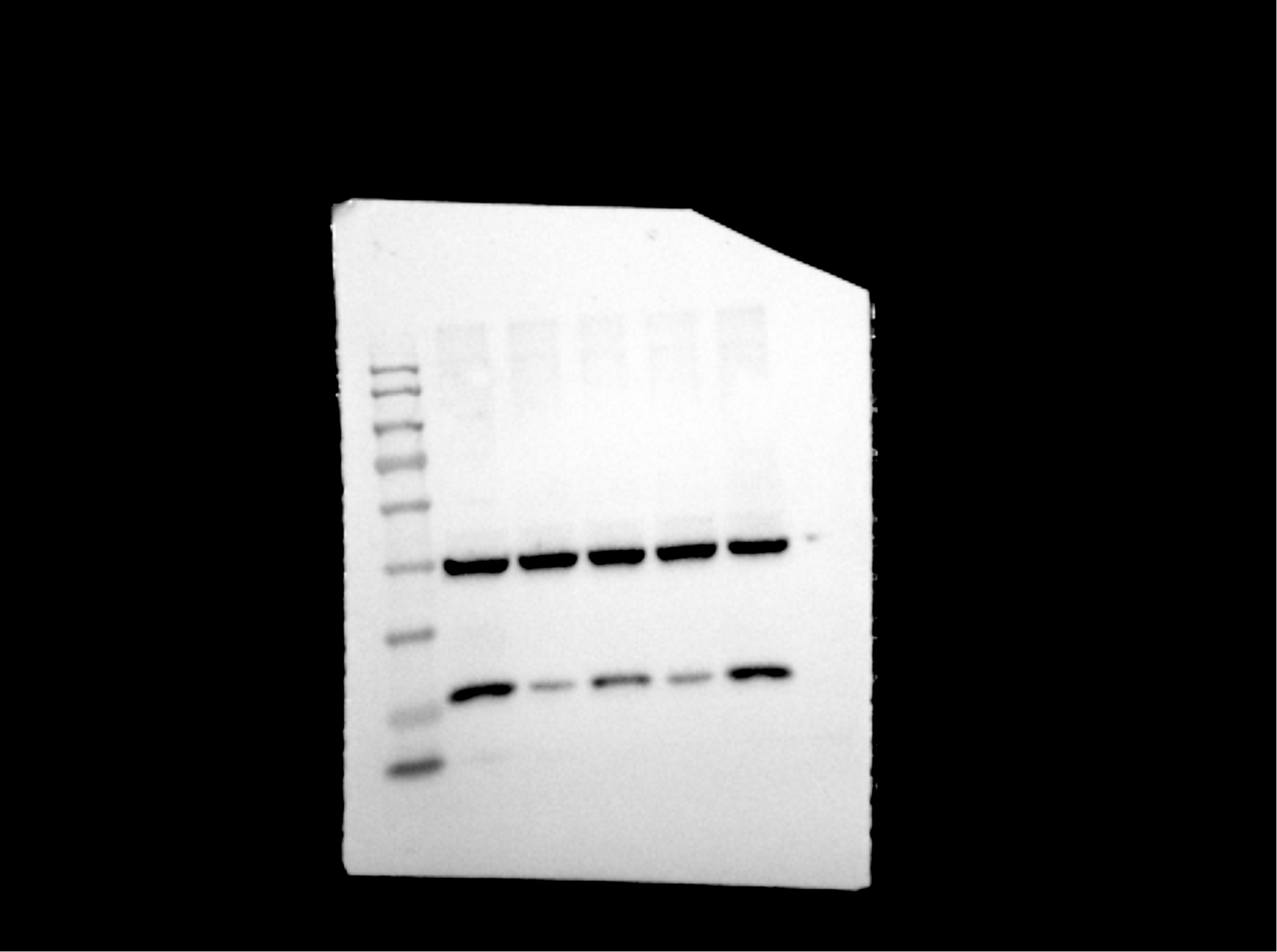

Supplement: Supplementary file 1 [file DataSheet3.ZIP › Full and uncropped western blots of Figure 6/Full and uncropped western blots of Figure 6D-2.jpg]

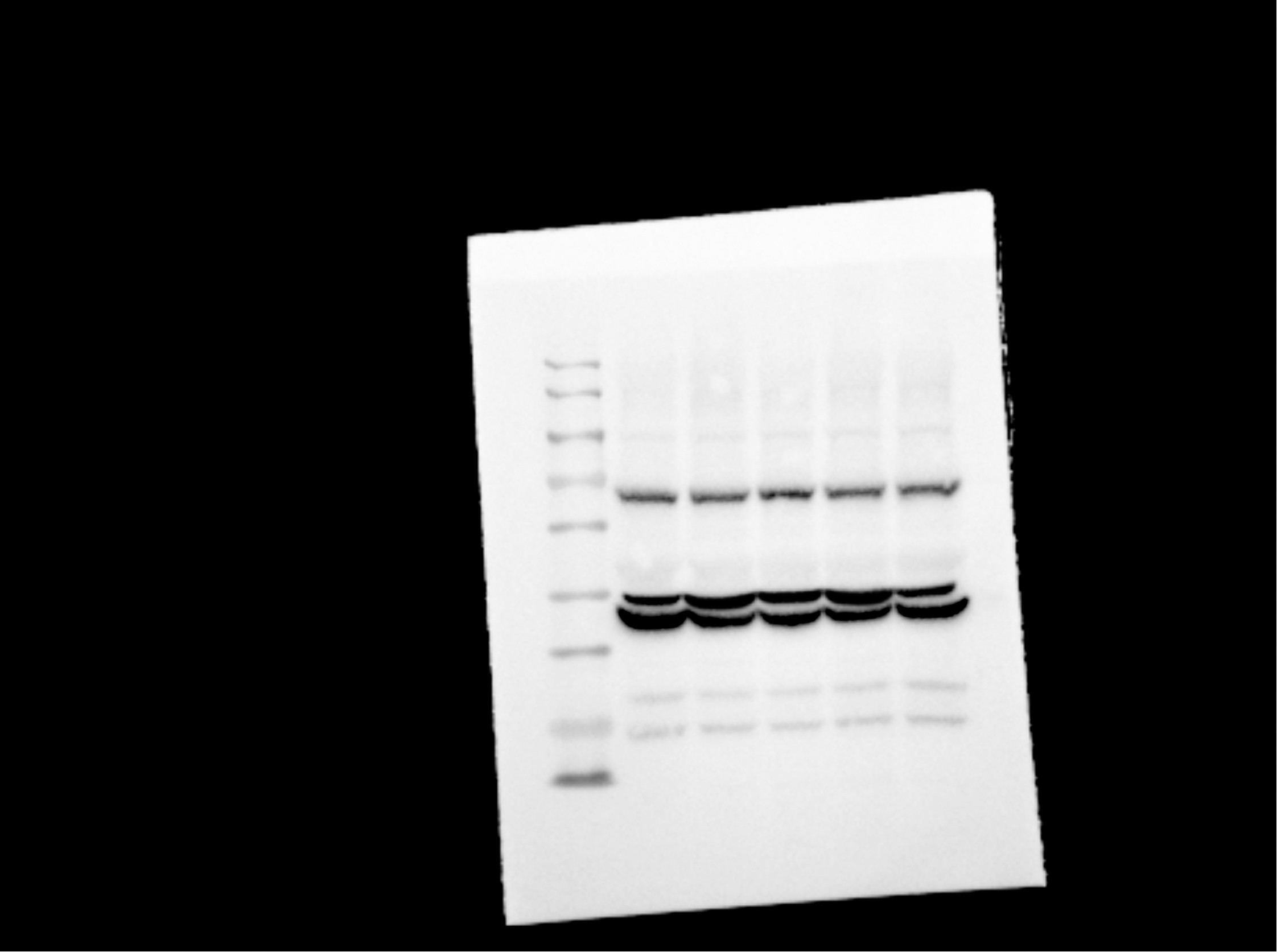

Supplement: Supplementary file 1 [file DataSheet3.ZIP › Full and uncropped western blots of Figure 6/Full and uncropped western blots of Figure 6D-3.jpg]

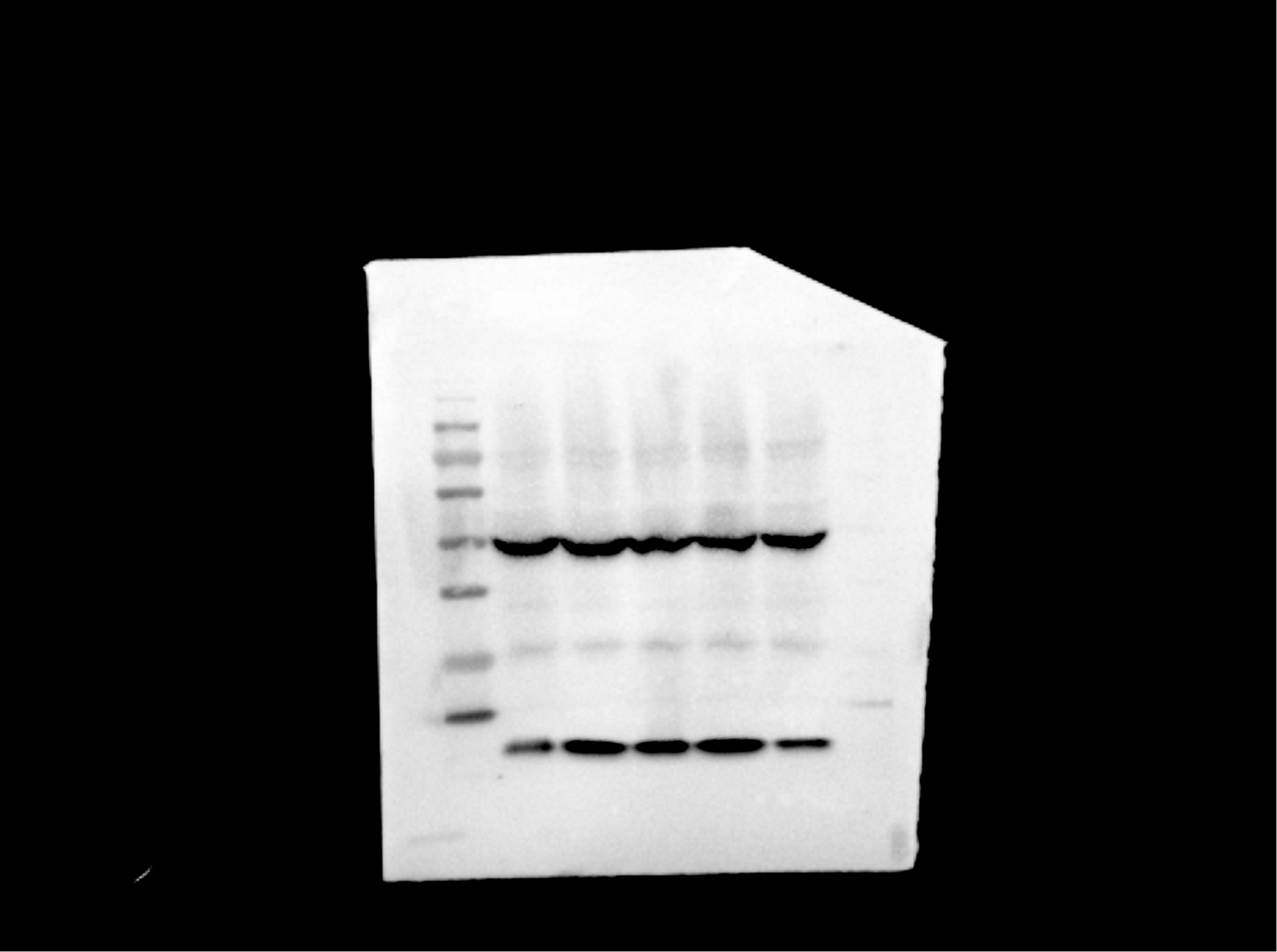

Supplement: Supplementary file 2 [file DataSheet4.ZIP › Full and uncropped western blots of Figure 7/Full and uncropped western blots of Figure 7B.jpg]

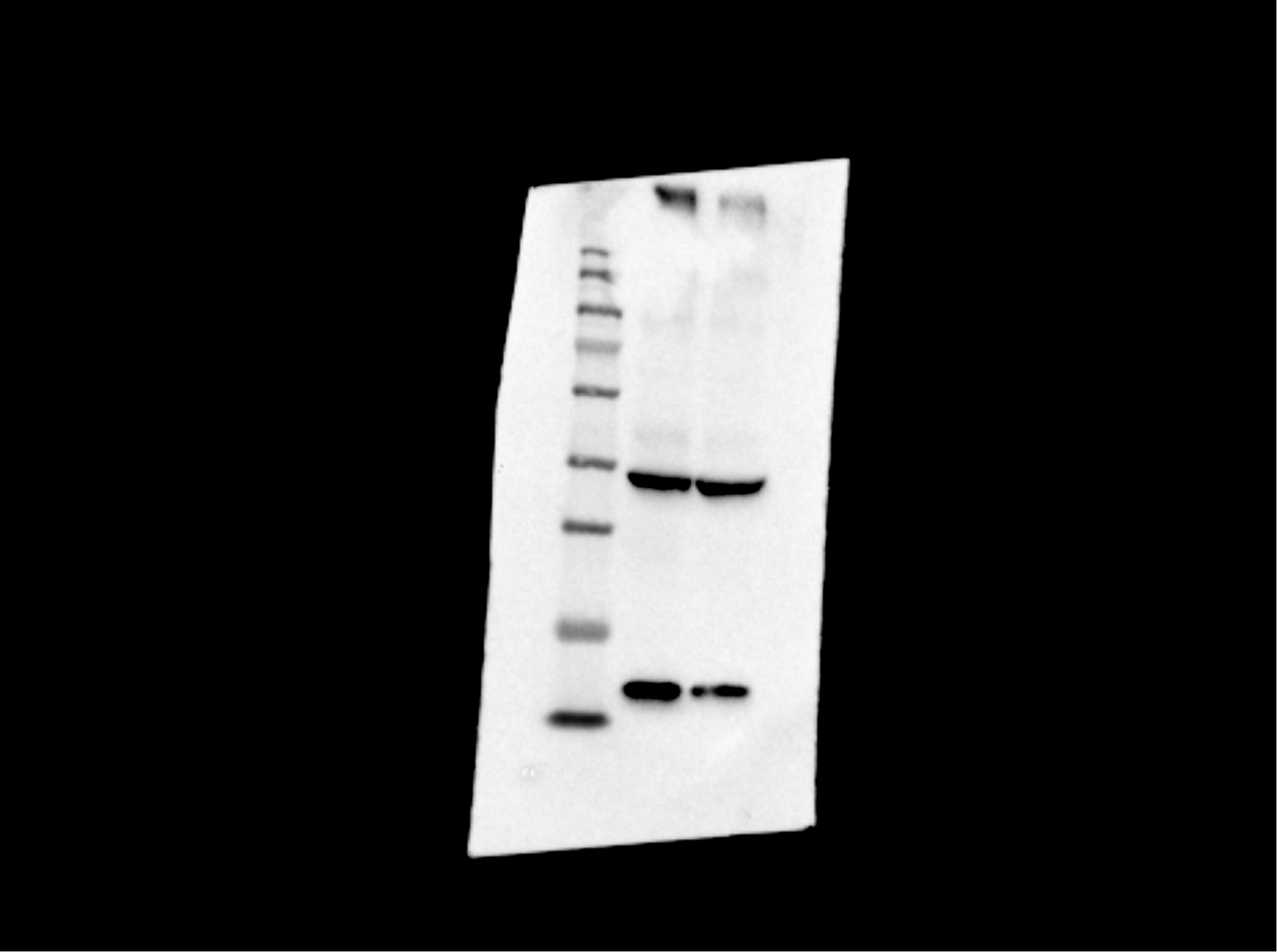

Supplement: Supplementary file 2 [file DataSheet4.ZIP › Full and uncropped western blots of Figure 7/Full and uncropped western blots of Figure 7D-1.jpg]

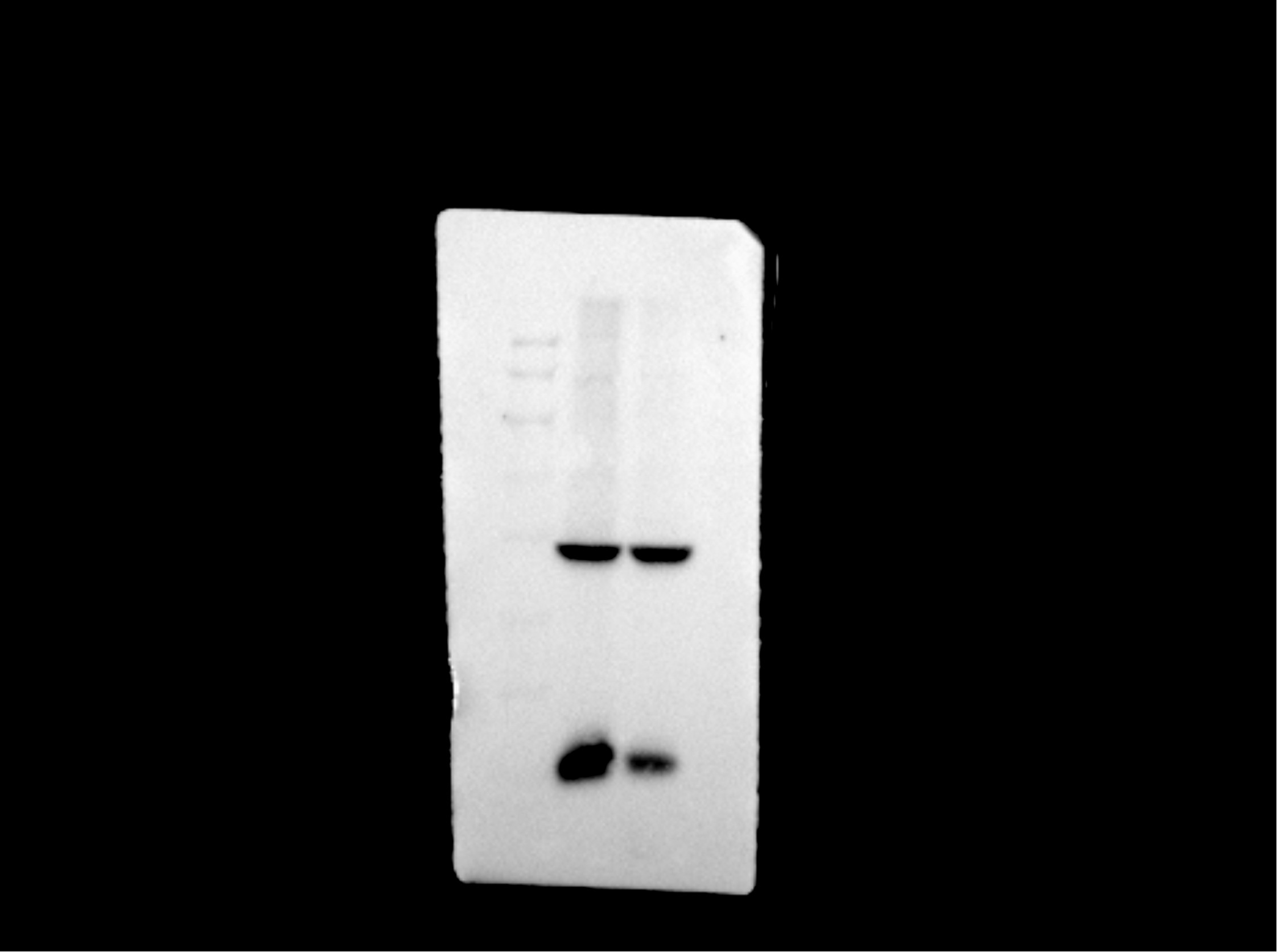

Supplement: Supplementary file 2 [file DataSheet4.ZIP › Full and uncropped western blots of Figure 7/Full and uncropped western blots of Figure 7D-2.jpg]

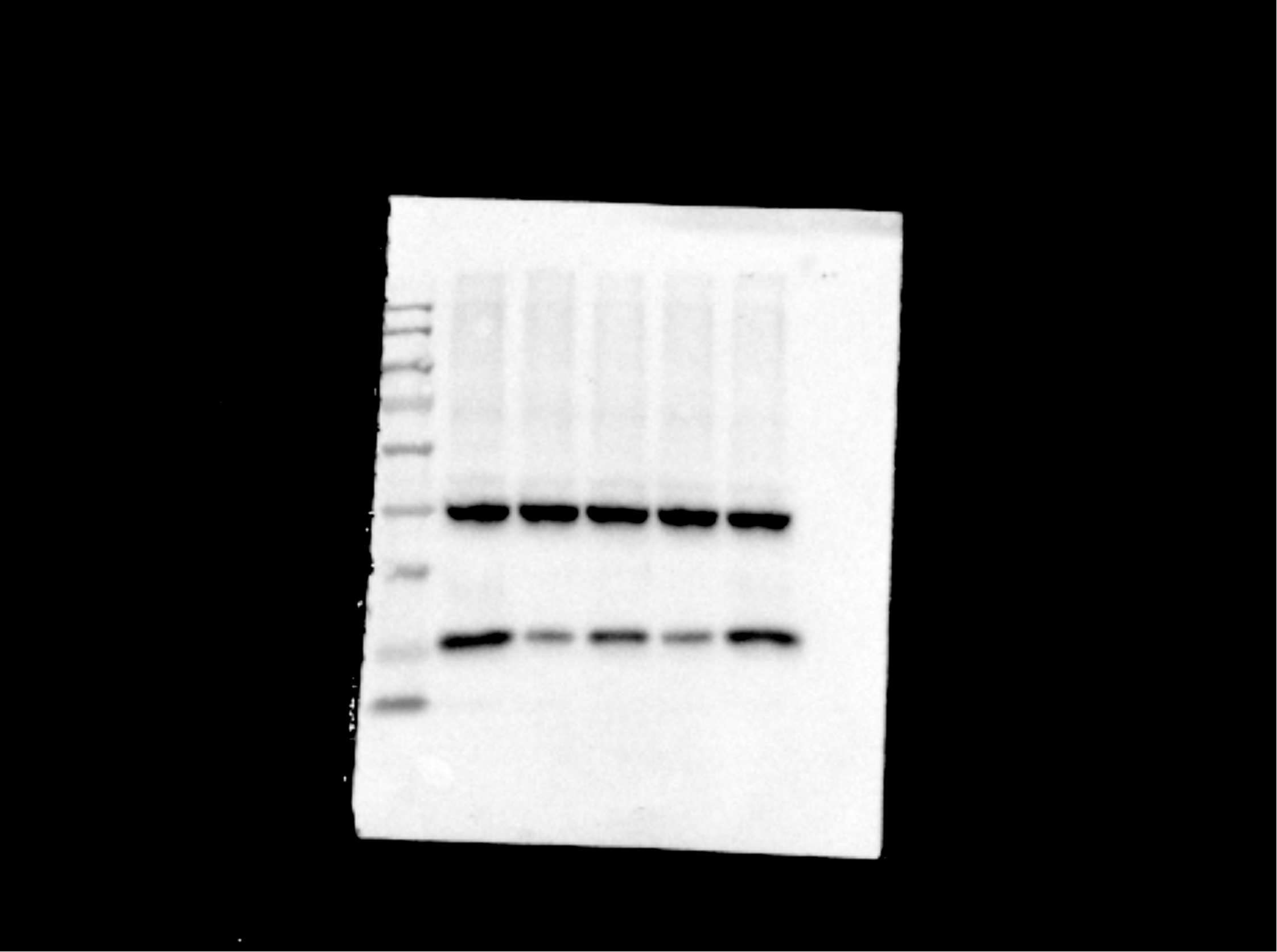

Supplement: Supplementary file 4 [file DataSheet2.ZIP › Full and uncropped western blots of Figure 5/Full and uncropped western blots of Figure 5E-1.jpg]

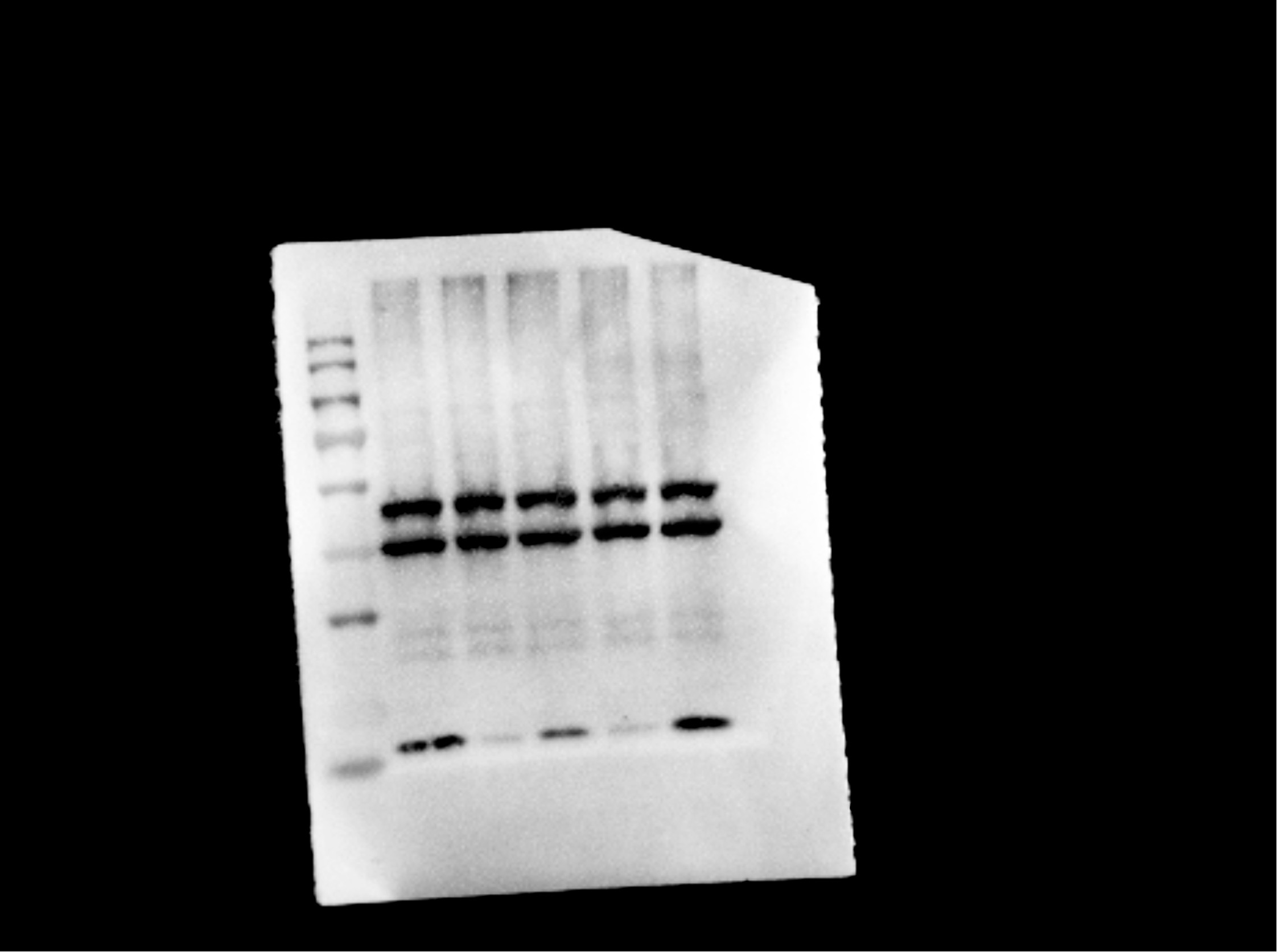

Supplement: Supplementary file 4 [file DataSheet2.ZIP › Full and uncropped western blots of Figure 5/Full and uncropped western blots of Figure 5E-2.jpg]

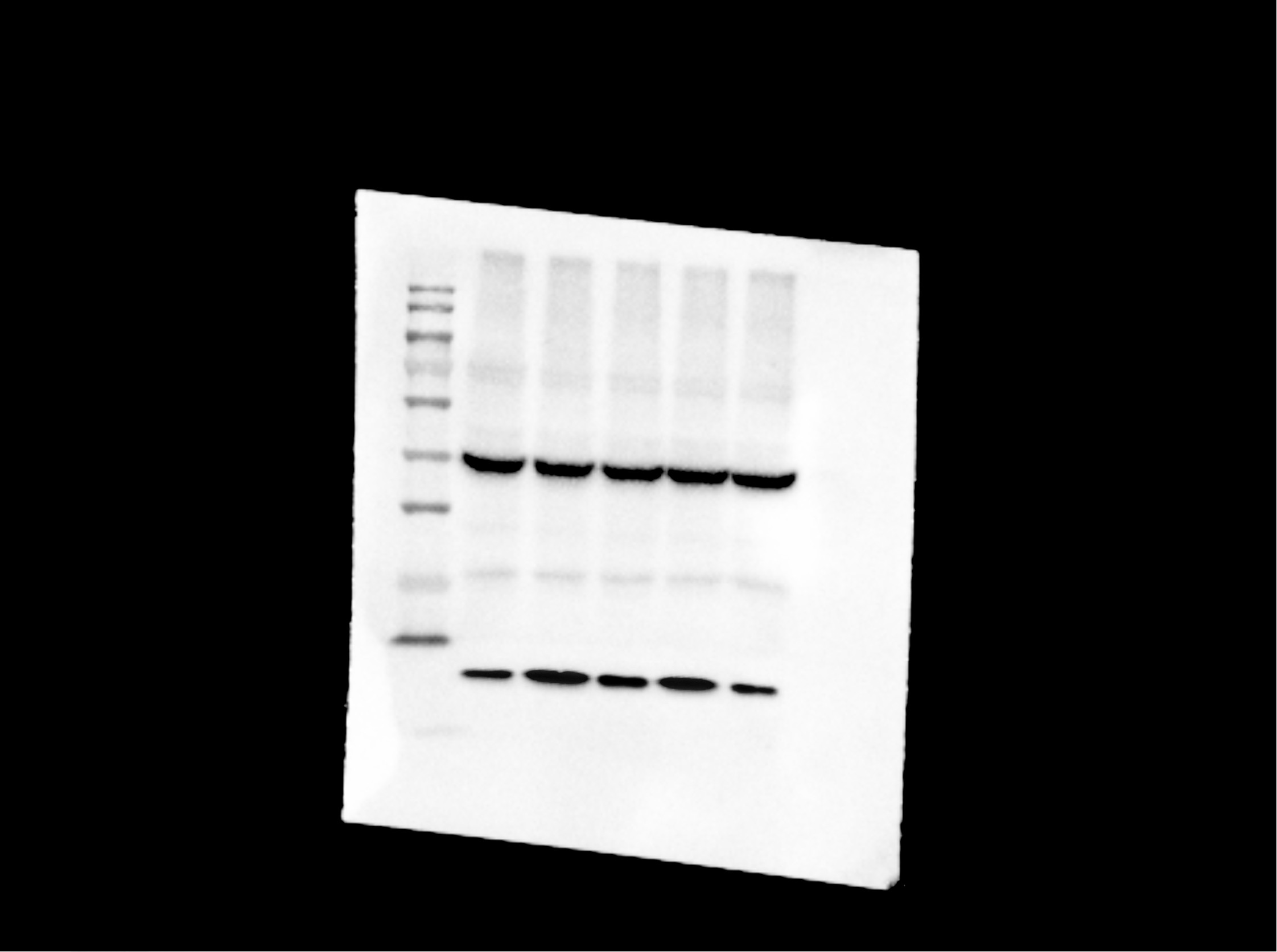

Supplement: Supplementary file 5 [file DataSheet5.ZIP › Full and uncropped western blots of Figure 8/Full and uncropped western blots of Figure 8B.jpg]

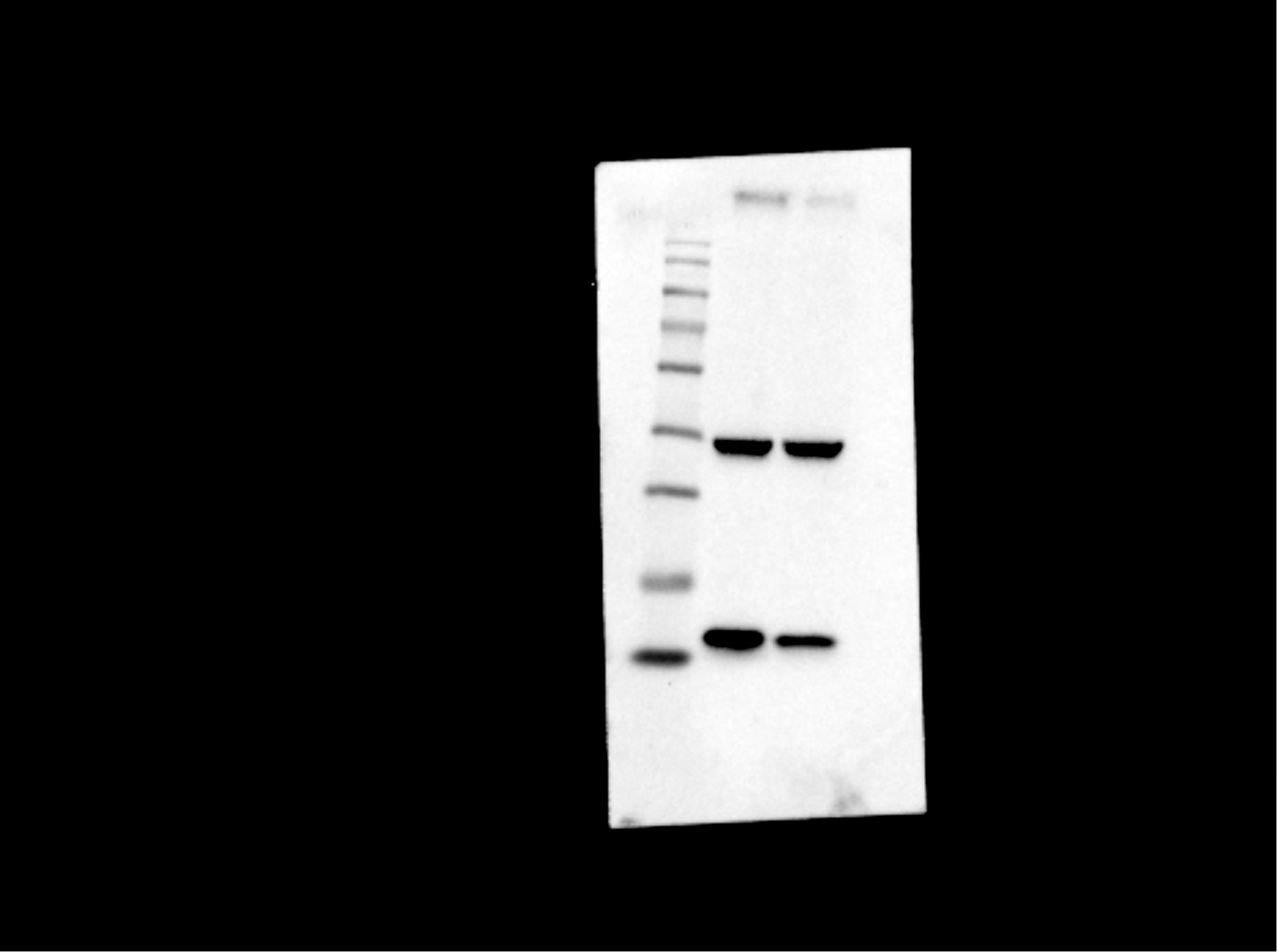

Supplement: Supplementary file 5 [file DataSheet5.ZIP › Full and uncropped western blots of Figure 8/Full and uncropped western blots of Figure 8H-1.jpg]

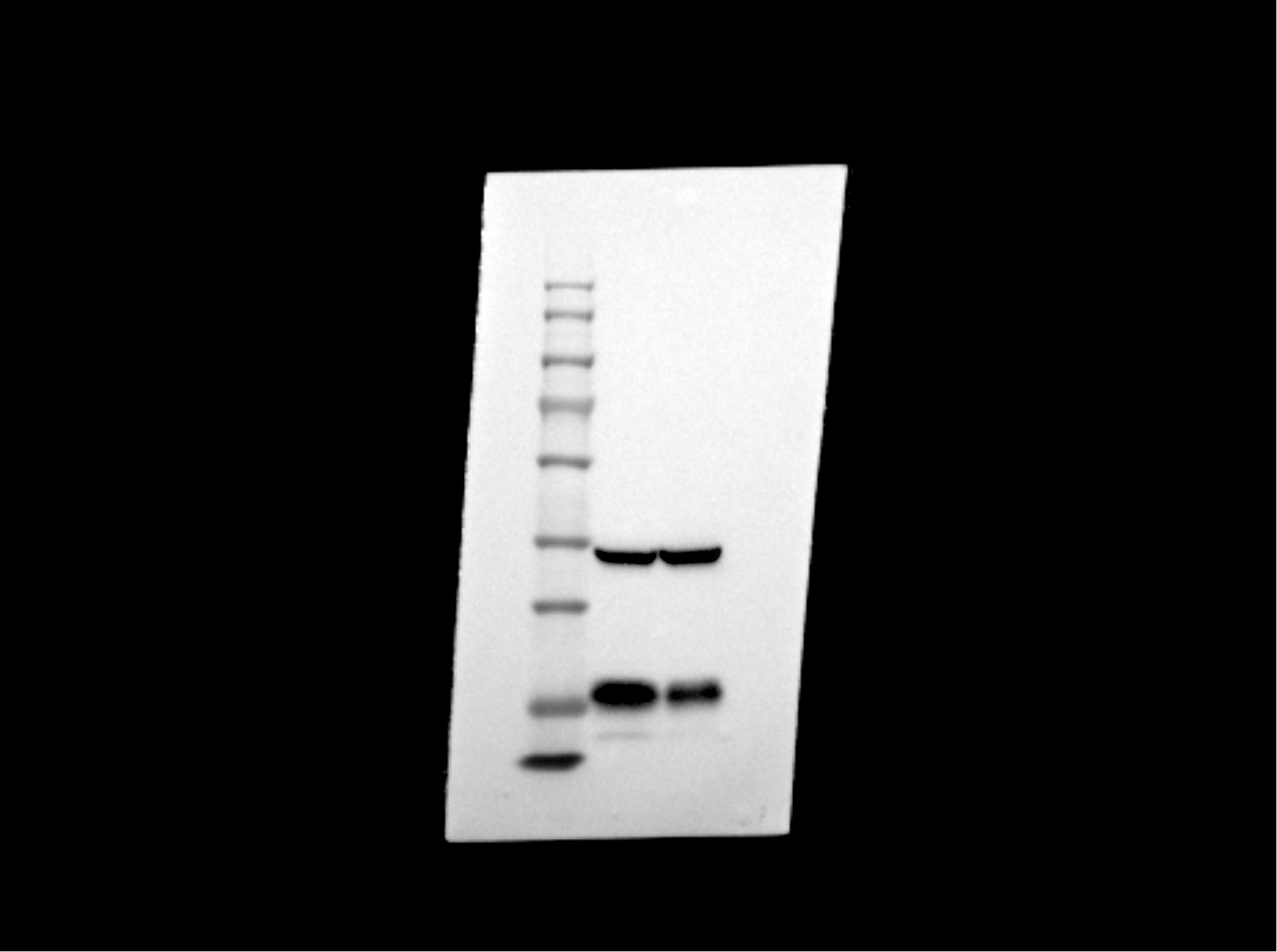

Supplement: Supplementary file 5 [file DataSheet5.ZIP › Full and uncropped western blots of Figure 8/Full and uncropped western blots of Figure 8H-2.jpg]
